# Iron Age and Anglo-Saxon genomes from East England reveal British migration history

**DOI:** 10.1038/ncomms10408

**Published:** 2016-01-19

**Authors:** Stephan Schiffels, Wolfgang Haak, Pirita Paajanen, Bastien Llamas, Elizabeth Popescu, Louise Loe, Rachel Clarke, Alice Lyons, Richard Mortimer, Duncan Sayer, Chris Tyler-Smith, Alan Cooper, Richard Durbin

**Affiliations:** 1 Wellcome Trust Sanger Institute, Hinxton, Cambridge CB10 1SA, UK; 2 Australian Centre for Ancient DNA, School of Biological Sciences and The Environment Institute, University of Adelaide, Adelaide, South Australia 5005, Australia; 3 Oxford Archaeology East, 15 Trafalgar Way, Bar Hill, Cambridge CB23 8SQ, UK; 4 Oxford Archaeology South, Janus House, Osney Mead, Oxford OX2 0ES, UK; 5 School of Forensic and Applied Sciences, University of Central Lancashire, Preston PR1 2HE, UK

## Abstract

British population history has been shaped by a series of immigrations, including the early Anglo-Saxon migrations after 400 CE. It remains an open question how these events affected the genetic composition of the current British population. Here, we present whole-genome sequences from 10 individuals excavated close to Cambridge in the East of England, ranging from the late Iron Age to the middle Anglo-Saxon period. By analysing shared rare variants with hundreds of modern samples from Britain and Europe, we estimate that on average the contemporary East English population derives 38% of its ancestry from Anglo-Saxon migrations. We gain further insight with a new method, rarecoal, which infers population history and identifies fine-scale genetic ancestry from rare variants. Using rarecoal we find that the Anglo-Saxon samples are closely related to modern Dutch and Danish populations, while the Iron Age samples share ancestors with multiple Northern European populations including Britain.

## Abstract (web summary)

This study examines ancient genomes of individuals from the late Iron Age to the middle Anglo-Saxon period in the East of England. Using a newly devised analytic algorithm, the author also estimate the relative ancestry of East English genome derived from Anglo-Saxon migrations and to the rest of Europe.

## Graphical abstract

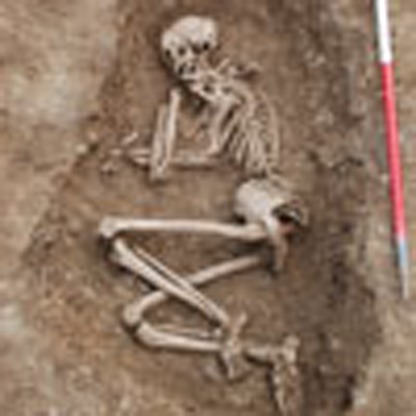

Within the last 2,000 years alone, the British Isles have received multiple well-documented immigrations. These include military invasions and settlement by the Romans in the first century CE, peoples from the North Sea coast of Europe collectively known as the Anglo-Saxons between ca. 400 and 650 CE (Fig. 1a), Scandinavians during the late Saxon ‘Viking period' 800–1,000 CE and the Normans in 1,066 CE (ref. 1). These events, along with prior and subsequent population movements, have led to a complex ancestry of the current British population. Although there is only a slight genetic cline from north to south at a coarse level^2^,^3^, recent analyses have revealed considerable fine-scale genetic structure in the Northern and Western parts of Great Britain, alongside striking homogeneity in Southern and Eastern England^4^ in the regions where archaeologists identify early Anglo-Saxon artifacts, cemeteries and communities. A variety of estimates of the fraction of Anglo-Saxon genetic ancestry in England have been given^5^,^6^,^7^,^8^, with the recent fine structure analysis suggesting most likely 10–40% (ref. 4).

However, even large-scale analyses of present-day data provide only weak evidence of the Anglo-Saxon migration impact, mainly for two reasons. First, estimating the impact of historical migrations from present-day genetic data alone is challenging, because both the state of the indigenous population before the migration as well as the genetic make up of the immigrants are unknown and have to be estimated simultaneously from present-day data. Second, if the source population is genetically close to the indigenous population, migrations are hard to quantify due to the challenge in detecting small genetic differences. This is particularly true for the case of the Anglo-Saxon migrations in Britain, given the close genetic relationships across Europe^9^,^10^.

Here we address both of these challenges using ancient DNA and new methodology. We present whole-genome sequences of 10 ancient samples from archaeological excavations in East England, which date to the late Iron Age and to the early and middle Anglo-Saxon periods and hence let us directly observe and quantify the genetic impact of the Anglo-Saxon migrations in England. Furthermore, we develop new methodology based on rare genetic variation in hundreds of modern samples to detect subtle genetic differentiation between immigrant and indigenous ancestry. We estimate that the modern-day East English population derives on average 38% of its ancestry from Anglo-Saxon migrants. We give evidence for mixing of migrants and natives in the early Anglo-Saxon period, and we show that the Anglo-Saxon migrants studied here have close ancestry to modern-day Dutch and Danish populations.

## Results

### Samples and sequencing

**Figure 1:**
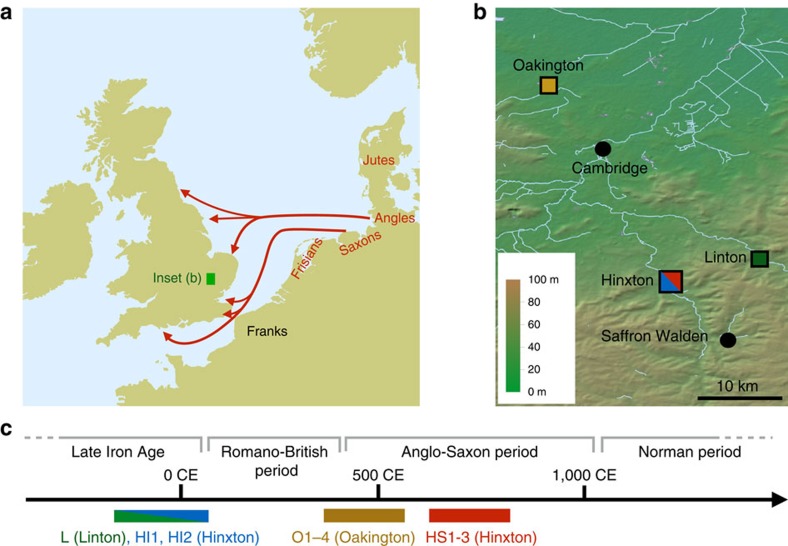
Geographic and temporal context of the samples used in this study. (**a**) Anglo-Saxon migration routes of people from the continental coast, as reconstructed from historical and archaeological sources. (**b**) The ancient samples used in this study were excavated at three archaeological sites in East England: Hinxton, Oakington and Linton. The towns Cambridge and Saffron Walden are also shown (black circles). Background green/brown shades indicate altitude. The colours of the four sample match the ones in **c** and Fig. 2. (**c**) The 10 ancient samples belong to three age groups. The sample from Linton and two samples from Hinxton are from the late Iron Age, the four Oakington samples from the early Anglo-Saxon period and three Hinxton samples are from the middle Anglo-Saxon period.

We generated genome sequences for 10 samples that were collected from three sites in East England close to Cambridge: Hinxton (five samples, Supplementary Fig. 1), Oakington (four samples, Supplementary Fig. 2) and Linton (1 sample), which were selected from a total of 23 screened samples based on DNA preservation (Fig. 1b, Table 1, Supplementary Table 1, Supplementary Note 1). All sequenced samples were radiocarbon dated (Supplementary Table 2), and fall into three time periods: the Linton sample and two Hinxton samples are from the late Iron Age (∼100 BCE), the four samples from Oakington from the early Anglo-Saxon period (fifth to sixth century), and three Hinxton samples from the middle Anglo-Saxon period (seventh to ninth century; Fig. 1c). The two Iron Age samples from Hinxton are male, all other samples are female, based on Y chromosome coverage and consistent with the archaeology. All samples were sequenced to genome-wide coverage from 1x to 12x (Table 1). All have contamination rates below 2%, as estimated both from mitochondrial DNA and from nuclear DNA (Supplementary Table 3, Supplementary Note 2). Mitochondrial and Y chromosome haplogroups of all samples are among the most common haplogroups in present-day North-Western Europe (Table 1)^11^,^12^ and in this case not informative for distinguishing immigrant versus indigenous ancestry.

We generated a principal component plot of the 10 ancient samples together with relevant European populations selected from published data^13^,^14^ (Supplementary Fig. 3). The ancient samples fall within the range of modern English and Scottish samples, with the Iron Age samples from Hinxton and Linton falling closer to modern English and French samples, whereas most Anglo-Saxon era samples are closer to modern Scottish and Norwegian samples. Overall, though, population genetic differences between these samples at common alleles are small.

### Estimating the Anglo-Saxon component in modern Britain

**Figure 2:**
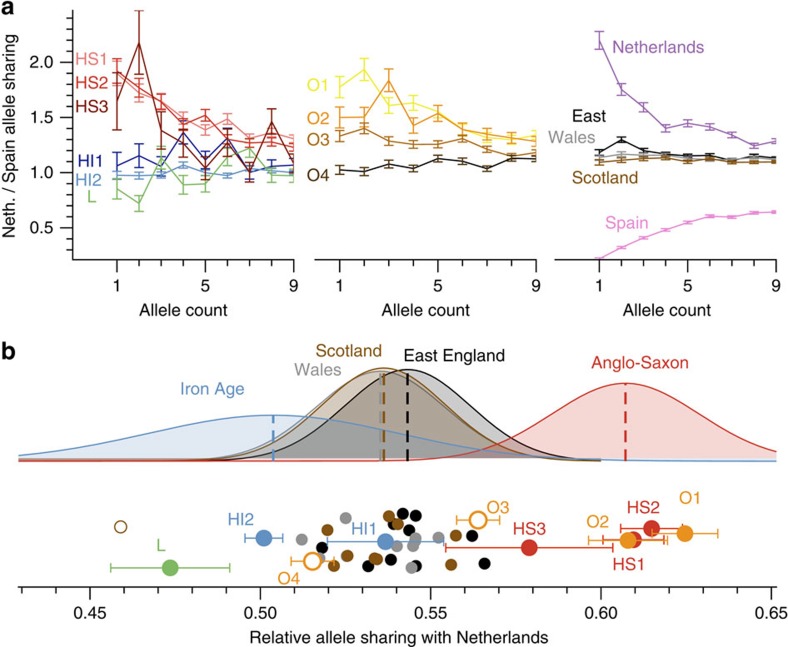
Relative rare allele sharing between ancient and modern samples. (**a**) The ratio of the numbers of rare alleles shared with modern Dutch and Spanish samples as a function of the allele count in the set of modern samples. Ancient sample codes (left-hand and middle sections) are defined in Table 1. Results from present-day British individuals (right hand panel) are averaged over 10 individuals from each subpopulation. Results from a Dutch and a Spanish individual are shown for comparison. Error bars are calculated from raw count statistics and using s.e. propagation (Methods section). (**b**) The relative fraction of rare alleles shared with modern Dutch compared with Spanish alleles, integrated up to allele count five in the modern samples. Iron Age and Anglo-Saxon samples mark the two extremes on this projection, while modern samples are spread between them, indicating mixed levels of Anglo-Saxon ancestry, which is on average higher in East England than in Wales and Scotland, with a large overlap. Two Early Anglo-Saxon samples from Oakington have been excluded from computing the average, indicated by empty circles, because they show evidence for being admixed (O3) or of non-immigrant ancestry (O4). One modern sample from Scotland is also excluded, indidated as empty circle because it is a clear outlier with respect to all other Scottish samples. Samples are shown with a random vertical offset for better clarity. Error bars (Methods section) for the modern samples are omitted here, but of the same order of magnitude as for the ancient samples. Data for this figure is available as Supplementary Data 1.

While principal component analysis can reveal relatively old population structure, such as generated from long-term isolation-by-distance models^15^, whole-genome sequences let us study rare variants to gain insight into more recent population structure. We identified rare variants with allele frequency up to 1% in a reference panel of 433 European individuals from modern Finland, Spain, Italy, Netherlands and Denmark, for which genome-wide sequence data are available^16^,^17^,^18^. We determined for each ancient sample the number of rare variants shared with each reference population (Supplementary Note 3). There are striking differences in the sharing patterns of the samples, illustrated by the ratio of the number of rare alleles shared with Dutch individuals to the number shared with Spanish individuals (Fig. 2a). The middle Anglo-Saxon samples from Hinxton (HS1, HS2 and HS3) share relatively more rare variants with modern Dutch than the Iron Age samples from Hinxton (HI1 and HI2) and Linton (L). The early Anglo-Saxon samples from Oakington are more diverse with O1 and O2 being closer to the middle Anglo-Saxon samples, O4 exhibiting the same pattern as the Iron Age samples, and O3 showing an intermediate level of allele sharing, suggesting mixed ancestry. The differences between the samples are highest in low-frequency alleles and decrease with increasing allele frequency. This is consistent with mutations of lower frequency on average being younger, reflecting more recent distinct ancestry, compared with higher frequency mutations reflecting older shared ancestry.

We also examined using the same method 30 modern samples from the UK10K project^19^, 10 each with birthplaces in East England, Wales and Scotland. Overall, these samples are closer to the Iron Age samples than to the Anglo-Saxon era samples (Fig. 2a). There is a small but significant difference between the mean values in the three modern British sample groups, with East English samples sharing slightly more alleles with the Dutch, and Scottish samples looking more like the Iron Age samples.

To quantify the ancestry fractions, we fit the modern British samples with a mixture model of ancient components, by placing all the samples on a linear axis of relative Dutch allele sharing that integrates data from allele counts 1–5 (Fig. 2b, Supplementary Note 3). By this measure the East England samples are consistent with 38% Anglo-Saxon ancestry on average, with a large spread from 25 to 50%, and the Welsh and Scottish samples are consistent with 30% Anglo-Saxon ancestry on average, again with a large spread (Supplementary Table 4). These numbers are lower on average if we exclude the low-coverage individual HS3 from the Anglo-Saxon group (35% for East English samples). A similar result is obtained when we analyse modern British samples from the 1,000 Genomes Project, which exhibit a strong substructure (Supplementary Note 4, Supplementary Fig. 4). We find that samples from Kent show a similar Anglo-Saxon component of 37% when compared against Finnish and Spanish outgroups, with a lower value for samples from Cornwall (Supplementary Fig. 5a, Supplementary Table 4).

An alternative and potentially more direct approach to estimate these fractions is to measure rare allele sharing directly between the modern British and the ancient samples. While being much noisier than the analysis using Dutch and Spanish outgroups, this yields consistent results (Supplementary Fig. 5b, Supplementary Note 3). In summary, this analysis suggests that on average 25–40% of the ancestry of modern Britons was contributed by Anglo-Saxon immigrants, with the higher number in East England closer to the immigrant source. The difference between groups within Britain is surprisingly small compared with the large differences seen in the ancient samples. This is true for both the UK10K samples and for the British samples from the 1,000 Genomes project, although we note that the UK10K sample locations may not fully reflect historical geographical population structure because of recent population mixing.

One caveat of our analysis is that we are using the three Iron Age samples from Cambridgeshire as proxies for the indigenous British population, which no doubt was structured, though it seems reasonable to take these as representatives at least for Eastern England. Furthermore, any continental genetic contribution from the Romano-British period would be factored into the assigned Anglo-Saxon component, as would a late Anglo-Saxon Scandinavian or Norman contribution. However these effects would only be strong if the contribution was large and heavily biased on the Dutch–Spanish axis.

### Building a population history model from rare variants

To get further insight into the history underlying these sharing patterns, we developed a sensitive new method, rarecoal, which fits a demographic model to the joint distribution of rare alleles in a large number of samples (Supplementary Notes 5 and 6). Our strategy is to build a model in the form of a population phylogeny of the relationship between modern European populations, into which we can place the ancient samples. We recognize that a model without admixture and post-split gene flow is inadequate as a complete description of European population history. However, this is a natural simplified model, and the focus in this study is on understanding the genetic relationships of immigrants and indigenous populations in England, for which this population phylogeny model provides a reasonable scaffold.

**Figure 3:**
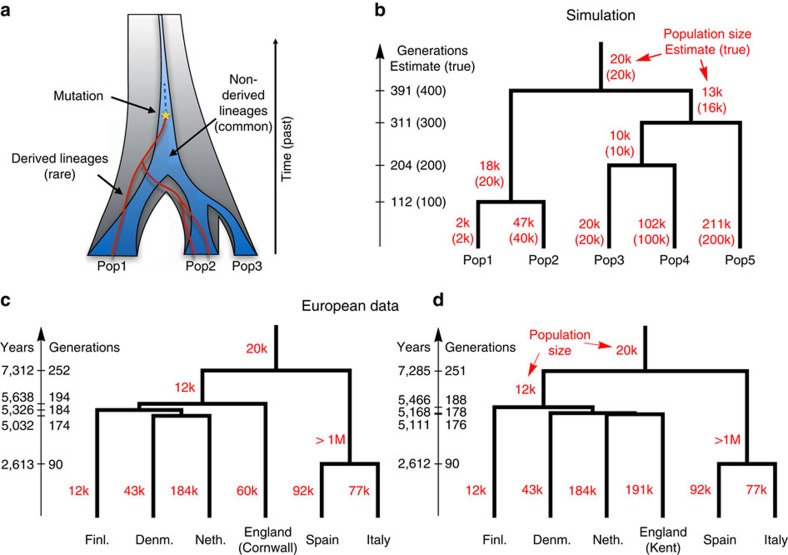
Modelling European history with rarecoal. (**a**) Rarecoal tracks the probabilities for the lineages of rare alleles (red) within a coalescent framework back in time, and approximates the distribution of non-derived alleles (dark blue) by its average. (**b**) By optimizing the likelihood of the data under the model, we can estimate population sizes and split times. Tested with simulated data, the estimates closely match the true values (in parentheses). (**c**) Applied to hundreds of European individuals, rarecoal estimates split times as indicated on the time axis and population sizes for each branch. (**d**) Same as **c**, but using samples from Kent instead of Cornwall as a proxy for the British population. The different tree topology between **c** and **d** reflects different population histories in Cornwall compared with Kent in the South of England.

The key idea is to model explicitly the uncertainty in the past of the distribution of derived alleles, but approximate the corresponding distribution for non-derived alleles by its expectation (Fig. 3a). Because rarecoal explicitly models rare mutations, it estimates separations in mutation clock time rather than genetic drift time, in contrast to methods based on allele frequency changes in common variants^20^. We first tested rarecoal on simulated data and found that it was able to reconstruct split times and branch population sizes with good accuracy (Fig. 3b), matching allele sharing almost exactly (Supplementary Fig. 6). We also tested its robustness with a smaller sample size in only one population (as in the Danish samples studied here), and under admixture (Supplementary Note 5, Supplementary Fig. 7).

We next applied rarecoal to 524 samples from six populations in Europe (Fig. 3c,d) to estimate a European demographic tree into which we could place the ancient samples. Because the British samples in the 1,000 Genomes Project fall into three distinct clusters, reflecting three sample locations (from Kent, Cornwall and the Orkney Islands, as part of the Peoples of the British Isles project^4^,^21^, Supplementary Note 4)^16^, we fitted different trees to these different groups (Supplementary Fig. 8). The common feature in all three trees is a first split between Southern and Northern Europe with a median time ∼7,000 years ago, followed by three more separations close in time ∼5,000 years ago between Netherlands, Denmark, Finland and Britain. Interestingly, when using the British samples from Cornwall, we obtained a tree where Cornwall forms an outgroup to the Dutch, Danish and Finnish population (Fig. 3c). In contrast, when we use Kent, it forms a clade with the Dutch population (Fig. 3d), consistent with higher Anglo-Saxon ancestry in the South of England than in Cornwall. When we use the Orkney population as the British branch, we find a similar tree topology as the one for Cornwall. These results show that both Cornwall and Orkney are more distantly related to continental Europe than Kent is. The tip branch effective population size is lowest in Finland (∼12,000), consistent with previous observations^22^,^23^, and highest in Kent (∼191,000) and in the Netherlands (∼184,000). For the European data, the allele sharing fit is worse than for the simulated data (Supplementary Fig. 9), presumably due to simplifying model assumptions of a constant population size in each branch and the absence of migration.

The relatively recent estimate for the split time between Italy and Spain, ∼2,600 years ago, may be a consequence of migration following an earlier separation; the population size of the Italian-Spanish ancestral population was estimated to be extremely large and an upper bound could not be determined, which could be an artifact of ancestral substructure or admixture. Another explanation would be a common source of admixture into both the Spanish and the Italian population, resulting in relatively recent common ancestry. We show in Supplementary Fig. 7 how admixture can modify rarecoal estimates of effective population size estimates and split times.

### Modelling ancestry of ancient genomes using rarecoal

**Figure 4:**
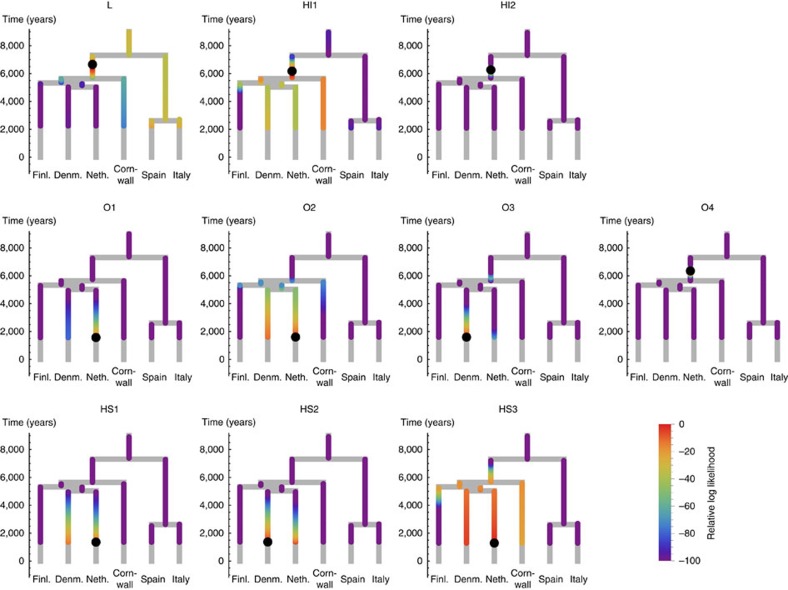
Placing ancient samples into the European tree. Given the European tree with Cornwall as British population branch, we map ancient samples onto this tree. We colour each point in the tree according to the likelihood that the ancestral branch of the ancient sample merges at that point. The maximum likelihood merge point is marked by a black circle. The analysis shows that Iron Age samples L, HI1 and HI2 have highest likelihood to merge onto the ancestral branch of all Northern European populations analysed, whereas the Anglo-Saxon samples merge into the Dutch and Danish branches, respectively. The low coverage samples L, HI1 and HS3 have the biggest spread in likelihood, but are consistent with the higher coverage samples.

In addition to reconstructing the broader European relationship from a large sample set, rarecoal can be used to evaluate the relationship of a single ancient sample with the European tree. To do this, we assume a model in which the ancestral population of the single sample merges with the European tree at a particular branch at a particular time before the date of origin of the sample. We can then use rarecoal to evaluate the likelihood of the joint allele sharing data between the ancient sample and the modern populations under each model, specified by the branch and merge time in the tree (Fig. 4, Supplementary Note 5). There was a marked difference between the Iron Age and the Anglo-Saxon era samples: the Anglo-Saxon era samples mostly merged onto the Dutch and Danish branches, whereas the Iron Age samples preferentially merged at the base of the ancestral branch for all modern Northern European samples. The exception is that the early Anglo-Saxon O4 shows the same signal as the Iron Age samples, consistent with the rare allele sharing analysis (Fig. 2). For sample O3, which appeared to be of mixed ancestry in the allele sharing analysis, we find highest likelihood for merging with the Danish branch. However, in this sample there is also a notably higher likelihood to merge onto the same Northern European ancestral branch point as seen for the Iron Age samples. This is consistent with O3 being of recently mixed indigenous and Anglo-Saxon origin, although we can not rule out more complex scenarios involving prior mixed ancestry of this individual during the Romano-British period. There is some differentiation amongst the Anglo-Saxon era samples with samples O1, O2, HS1 and HS3 having highest likelihood of merging onto the Dutch branch while O3 and HS2 have highest likelihoods of merging onto the Danish branch, although in some cases the difference in likelihood between these two possibilities is small. The signals from HS3, HI1 and L are more spread due to low coverage, but consistent with the other results.

The mapping of the ancient samples onto the tree is similar for the tree using Kent as British population (Supplementary Fig. 10) and for the tree using Cornwall as the British proxy (Fig. 4). In particular, the Iron Age samples map onto the ancestral branch of Northern European populations irrespective of using Kent or Cornwall as British proxy. This suggests that none of the present-day populations in our data set, including the population from Cornwall, are as closely related to the Iron Age samples as Denmark and the Netherlands are to the Anglo-Saxon samples.

We validated our approach of mapping individual samples into a tree by placing modern samples onto the same tree as in Fig. 4. We find all samples from populations used in building the tree placed on the tip of their respective branch as expected (Supplementary Fig. 11). When mapping samples from groups not present in the tree, as is the case for samples from Kent and Orkney, we find that they map onto the same ancestral location as the Iron Age samples (Supplementary Fig. 11), confirming that they are of distinct ancestry from the Cornish population and other populations used in building the tree, similarly to the Iron Age samples. As detailed in Supplementary Note 5, our mapping approach crucially depends on an appropriate model for the reference populations. When using the Kent population for building the tree (Fig. 3c), we find that mapping British samples becomes worse (Supplementary Fig. 12), arguably because the Kent population is less genetically defined and more admixed than the group from Cornwall. In such cases we need to model population phylogenies with admixture and gene flow, and further development on rarecoal will enable us to study these more complex scenarios.

## Discussion

This study combines large modern sample sets with ancient genomes in a novel way, based on rare allele sharing. On the one hand, the power of rare genetic variants clearly shows the value in whole-genome sequencing of ancient DNA: While SNP capture technology provides a far more economical way to obtain genome-wide data from ancient DNA (ref. 14), it cannot detect rare genetic variants, which as we have shown are necessary to analyse subtle genetic differences between closely related populations. On the other hand, our analysis shows the value of having whole-genome sequence for a large number of modern samples to ascertain rare variants, which fortunately is increasingly becoming the standard for large population scale genetic studies^16^,^17^,^18^,^19^.

Our analysis of early and middle Anglo-Saxon samples from East England adds significantly to our picture of the Anglo-Saxon period in Britain. In the cemetery at Oakington we see evidence even in the early Anglo-Saxon period for a genetically mixed but culturally Anglo-Saxon community^24^,^25^, in contrast to claims for strong segregation between newcomers and indigenous peoples^7^. The genomes of two sequenced individuals (O1 and O2) are consistent with them being of recent immigrant origin, from a source population close to modern Dutch, one was genetically similar to native Iron Age samples (O4), and the fourth was consistent with being an admixed individual (O3), indicating interbreeding. Despite this, their graves were conspicuously similar, with all four individuals buried in flexed position, and with similar grave furnishing. Interestingly the wealthiest grave, with a large cruciform brooch, belonged to the individual of native British ancestry (O4), and the individual without grave goods was one of the two genetically ‘foreign' ones (O2), an observation consistent with isotope analysis at West Heslerton which suggests that new immigrants were frequently poorer^26^,^27^.

Up to this point we have interpreted the genetic structure of the Anglo-Saxon samples in terms of recent immigrant versus indigenous populations. However, in the absence of a time series through the Romano-British period from the Iron Age to the Anglo-Saxon period, we should also consider the possibility that some of the genetic heterogeneity seen in the Oakington samples arose earlier due to immigration in Romano-British times. We recall that sample O4 lies genetically almost centrally in the Iron Age samples, and O1 and O2 are very close to the later Middle Saxon samples from Hinxton and modern Dutch. For Roman immigration patterns to generate this diverse structure in the fifth to sixth century Oakington samples, one would have to assume strong social segregation with little interbreeding over multiple generations. This seems unlikely given that immigration into Roman-Britain was geographically diverse and consisted of an administrative elite^28^ and the military, who would have interbred and recruited locally, particularly in the last decades of the third and fourth centuries^29^. Furthermore, there is no significant Roman settlement at Oakington and no evidence for significant Roman Heritage^30^.

Given the mixing apparent ∼500 CE, and that the modern population is not more than 40% of Anglo-Saxon ancestry, it is perhaps surprising that the middle Anglo-Saxon individuals from the more dispersed field cemetery in Hinxton look more genetically consistent with unmixed immigrant ancestry. One possibility is that this reflects continued immigration until at least the Middle Saxon period. The unmixed Hinxton group, versus the mixing of the Oakington population, shows that early medieval migration took a variety of forms and that these migrants integrated with the incumbent population in different ways. Full-genome sequences, and new methods such as rarecoal, now allow us to use slight distinctions in genetic ancestry to study such recent events. Further ancient genomes, and methodological improvements to incorporate explicit migration and mixing, will enable us to resolve them in more detail.

## Methods

Custom software mentioned here is publically available on www.github.com/stschiff/sequenceTools and www.github.com/stschiff/rarecoal.

### DNA extraction

Samples were first treated with UV-light (260 nm) for 20–30 min, and the surface was cleaned with bleach (3.5%) and isopropanol. The sample surface was mechanically removed using a Dremel drill and disposable abrasive discs. Samples were ground to fine powder using a Mikrodismembrator (Sartorius) and stored at 4**°**C until further use. DNA was extracted in clean room facilities in Adelaide using an in-solution silica-based protocol^31^.

### Library preparation

Libraries were generated from the Hinxton individuals (*n*=6) with^32^ and without enzymatic damage repair (Supplementary Table 1), whereas partial damage repair^33^ was performed for the Linton (*n*=3) and Oakington (*n*=14) samples. All 29 libraries were prepared with truncated barcoded Illumina adaptors and amplified with full-length indexed adaptors for sequencing^34^. Protocols evolved over the course of the study with regards to the final library amplification steps. Hinxton DNA libraries were amplified by PCR in quintuplicates for an initial 13 cycles (AmpliTaq Gold, Life Technologies), followed by pooling and purification of the PCR replicates with the Agencourt AMPure XP system. DNA libraries were then re-amplified for another 13 cycles in quintuplicates or sextuplicates, followed by pooling and purification, visual inspection on a 3.5% agarose gel, and final quantification using a NanoDrop 2000c spectrophotometer (FisherScientific). The Oakington and Linton DNA libraries were amplified using isothermal amplifications using the commercial TwistAmp Basic kit (TwistDx Ltd). The amplification followed the manufacturer's recommendations and used 13.4 μl of libraries after the Bst fill-in step, and an incubation time of the isothermal reaction of 40 min at 37 °C, followed by gel electrophoresis and quantification using a Nanodrop spectrophotometer. Following quantification, libraries were re-amplified for seven cycles using full-length 7-mer indexed Illumina primers as described^34^, followed by purification with Ampure and quantification using a TapeStation (Agilent).

### Library screening

The 23 libraries treated with damage repair were screened for complexity and endogenous DNA on an Illumina MiSeq platform in Harvard in collaboration with David Reich (Supplementary Table 1). When the project started, we had available only the samples from Hinxton, and since all of them had high complexity and high amounts of endogenous DNA (except 12882A, which did not pass screening), we selected all five samples for deep sequencing. We then expanded the project to the other two sites, from which we screened many more samples than we could sequence deeply, so we selected the best four samples (with highest complexity and endogenous DNA) from Oakington and the best from Linton (from which we had fewer samples, and there was only one sample with acceptable complexity for deep sequencing).

### Deep sequencing

We first sequenced the five DNA libraries generated from the Hinxton samples in two batches. The first batch consisted of 10 lanes of 75 bp paired end sequencing on an Illumina HiSeq 2500 platform, run in rapid mode. All five samples were multiplexed in this batch. The resulting data was processed (see below) and used to estimate complexity and endogenous DNA to decide further sequencing. The second batch consisted of 42 lanes with similar settings as the first batch, but not multiplexed. Based on the complexity and endogenous DNA estimates, we sequenced sample HI1 and HS3 on 4 lanes each, samples HS1 and HS2 on 8 lanes each and sample HI2 on 16 lanes. In the second batch, we introduced five dark cycles into read 1 to avoid low-complexity issues due to the clean room tags in the library preparation. We also included 5% Phi X sequences to increase the complexity of the first five base pairs of read 2, a common procedure for low-complexity libraries. In case of the samples from Oakington and Linton, we used the protocol used in batch 2 of the Hinxton samples (including dark cycles). We sequenced samples O2 and L on 4 lanes each, sample O4 on 6 lanes, sample O1 on 8 lanes and sample O3 on 10 lanes.

### Raw read processing

We filtered out all read pairs that did not carry the correct clean room tags in the first five base pairs of read 2. In case of batch 1 of the Hinxton samples, we also sequenced the clean room tag on read 1, which we also filtered on in these cases. As a second step, we merged all reads searching for a perfect or near perfect overlap allowing at most 1 mismatch between read 1 and the reverse complement of read 2. The merging also took advantage of the fact that we typically had fragments of length 50 pb, which means that many of the 75 bp reads contained the reverse complement of the clean room tag of the other read, and the Illumina adaptors. As a last step, we removed the clean room tags and the adaptors from both ends of the merged reads. Both merging and adaptor trimming was done using a custom programme called filterTrimFastq, available on http://www.github.com/stschiff/sequenceTools.

### Alignment

After merging, we ended up with single reads with variable length (on average about 50 bp) for each sample. We aligned those single reads with the programme ‘bwa aln'^35^ to the human reference, version GRCh37 using the parameter ‘-l 1024' to turn-off seeding^36^. The alignment was done on a per-lane basis, all alignments were then sorted using ‘samtools sort'. For each individual, we then merged the sorted alignments into a single bam file per individual, using ‘samtools merge'. We then removed duplicate reads in each alignments using our custom python script ‘samMarkDuplicates.py', available also on github. The script checks whether neighbouring reads in the sorted alignments are equal, and removes all but one read if it finds duplicates. Finally, we removed all unmapped reads from the alignments. Despite enzymatic damage repair, some low levels of DNA damage can still be found in the libraries. We used the programme ‘mapdamage2' (ref. 37) to measure DNA degradation. For each individual, we first ran mapDamage on chromosome 20 to estimate the degradation profile. For all individuals, the DNA damage profile was found to have an excess of C->T changes at the 5′ end of reads, as expected for ancient DNA, and an excess of G->A changes was found at the 3′ end. However, because the sequencing libraries were treated with UDG, which removes damaged sites in reads, the excess was much lower than in comparable studies without UDG treatment^37^.

### Mitochondrial and Y chromosome analysis

We called mtDNA and Y chromosome consensus sequences using samtools. Haplogroups were handcurated using public databases (Supplementary Note 2).

### Contamination estimates

We estimated possible modern DNA contamination in all ancient samples using two methods. First, we tested for evidence for contaminant mitochondrial DNA^38^. We looked for sites in the mitochondrial genome, at which the ancient sample carried a consensus allele that was rare in the 1,000 Genomes reference panel. We then looked whether there were reads at these sites that carried the majority allele from 1,000 Genomes (Supplementary Note 2). Second, we used the programme ‘verifyBamId'^39^ to carry out a similar test in the nuclear genome, again using the 1,000 Genomes reference panel. Contamination estimates are summarized in Supplementary Table 3.

### Principal component analysis

We downloaded the Human Origins Data set^13^,^14^ and called genotypes at all sites in this data set for all ancient samples using a similar calling method as described in ref. 14: Of all high-quality reads covering a site, we picked the allele that is supported by the majority of reads, requiring at least two reads supporting the majority allele, otherwise we call a missing genotype. If multiple alleles had the same number of supporting reads, we picked one at random. Principal component analysis was performed using the ‘smartpca‘ programme from EIGENSOFT (ref. 40), by using only the modern samples for defining the principal components and projecting the 10 ancient samples onto these components (Supplementary Fig. 3).

### Rare allele sharing analysis

We compiled a reference panel consisting of 433 individuals from Finland (*n*=99), Spain (*n*=107), Italy (*n*=107), Netherlands (*n*=100) and Denmark (*n*=20). The Finnish, Spanish and Italian samples are from the 1,000 Genomes Project (phase 3)^16^, the Dutch samples from the GoNL project^17^ and the Danish samples from the GenomeDK project^18^. For the Dutch and Danish samples, only allele frequency data was available. In case of the Dutch data set, we downsampled the full data set to obtain the equivalent of 100 samples. All other reference sample variant calls were used as provided by the 1,000 Genomes Project. In addition, we filtered based on a mappability mask^41^,^42^ that is available from www.github.com/stschiff/msmc. We selected all variants up to allele count nine in this reference set and tested for each ancient individual and each of those sites whether the ancient individual carried the rare allele. We called a rare variant (always assumed heterozygous) in the ancient sample if at least two reads supported the rare allele from the reference set. While this calling method will inevitably miss variants in low coverage individuals, the relative numbers of shared alleles with different populations is unbiased.

We accumulated the total number of alleles shared between each ancient sample and each modern reference population, and stratified by allele count in the reference population, up to allele count nine (Supplementary Data 1). We found that sharing with the Dutch and the Spanish population showed the largest variability across the ancient samples. For the plot in Fig. 2a, we divided the sharing count with the Dutch population by the sharing count of the Spanish population for each allele frequency. To plot curves from the Dutch and the Spanish population itself, we sampled haploid individuals from each population by sampling with replacement at every variant site in the reference set. This was necessary because for the Dutch samples no genotype information was publically available, only allele frequency data (Supplementary Note 3).

For the 30 UK10K samples shown in Fig. 2a,b, we started from the read alignment for each individual and called rare variants with respect to the 433 reference individuals in exactly the same way as we did for the ancient samples. For Fig. 2a, the allele sharing counts were then accumulated across the 10 individuals in each group. Error bars for each allele sharing count are based on the square root of each count. For Fig. 2b we added the allele sharing counts between each ancient sample and each reference population up to allele count five, and computed the ratio *NED/(NED+IBS)*, where *NED* is the sharing count with Dutch, and *IBS* the sharing count with Spanish (Supplementary Note 3). For the mean and variances shown in Fig. 2b, we excluded outliers as indicated in the caption of the figure. The fraction of Anglo-Saxon derived ancestry is computed for each modern UK10K sample as the relative distance of its relative sharing ratio from the Iron Age mean value compared with the Saxon era mean value, as shown in Fig. 2b, with 0% corresponding to the Iron Age mean, and 100% corresponding to the Anglo-Saxon era mean (Supplementary Note 3, Supplementary Table 4).

### Rarecoal analysis

Rarecoal is a new framework to calculate the joint allele frequency spectrum across multiple populations using rare alleles. Given a certain distribution of rare derived alleles across subpopulations (here up to allele count four), and a given number of non-derived alleles, which can be arbitrarily large, we calculate the total probability of that configuration under a demographic model. The model consists of a population tree with constant population sizes in each branch of the tree and split times. To give rise to the data observed in the present, the lineages of the derived alleles must coalesce among each other before they coalesce to any non-derived lineage. We introduce a state space that contains all possible configurations of derived lineages across populations and propagate a probability distribution over this space back in time. Details and mathematical derivations are given in Supplementary Note 6.

We implemented rarecoal in a software package (available from www.github.com/stschiff/rarecoal) that can learn the parameters of a given population tree topology from the data using numerical maximization of the likelihood and subsequent Markov Chain Monte Carlo to get posterior distributions for each split time and branch population size. We did not implement an automated way to learn the tree topology itself, but use a step by step protocol to learn the best topology fitting the data, adding one population at a time (Supplementary Note 5). The outputs from rarecoal are in scaled time. To convert to real time (years) and real population sizes, we used a per-generation mutation rate of 1.25 × 10^−8^ and a generation time of 29 years.

We tested the method on simulated data using the sequential coalescent with recombination model (SCRM) simulator^43^ with the model shown in Fig. 3b with 1,000 haploid samples distributed evenly across the five populations and realistic recombination and mutation parameters. We then learned the model from the European data set as shown in Fig. 3c using an iterative protocol, adding one population at a time and maximizing parameters subsequently to ensure that we are still fitting the right topology (Supplementary Note 5).

For mapping ancient samples on the tree we used the same calling method as in the rare allele sharing analysis. We then added the ancient individual as a separate seventh population to the European tree and evaluated the likelihood for this external branch to merge anywhere on the tree. We restricted the fitting to alleles that were shared with the ancient sample and excluded private variants in the ancient sample, which have high false-positive rates. We also made sure that the age of the ancient sample was correctly modelled into the joint seven-population tree, by ‘freezing' the state probabilities from the present up to the point where the ancient sample lived.

For testing the tree-colouring method, we used single individuals from within the reference set and used them as separate sample to be mapped onto the European tree. (Supplementary Note 5).

## Additional information

**Accession codes**: The raw sequence data of the 10 samples presented in this paper are deposited at the European Nucleotide Archive (http://www.ebi.ac.uk/ena). The study IDs are ERP003900 (Hinxton samples) and ERP006581 (Oakington and Linton samples).

**How to cite this article:** Schiffels, S. *et al.* Iron Age and Anglo-Saxon genomes from East England reveal British migration history. *Nat. Commun.* 7:10408 doi: 10.1038/ncomms10408 (2016).

## Supplementary Material

Supplementary Information Supplementary Figures 1-12, Supplementary Tables 1-4, Supplementary Notes 1-6 and Supplementary References

Supplementary Data 1 Data file containing the allele sharing counts of modern and ancient English samples, as shown in Figure 2

## Figures and Tables

**Table 1:** A summary of all sequenced samples in this study.

**Name**	**Origin**	**Sex**	**C14 Date (calibrated)**	**Endogenous (%)**	**Unique (%)**	**MT and Y haplogroup**	**Mean autosomal coverage**
L	Linton	Female	360–50 BCE	72	54	H1e	1.4
HI1	Hinxton	Male	160 BCE–26 CE	16	63	K1a1b1b, R1b1a2a1a2c	1.3
HI2	Hinxton	Male	170 BCE–80 CE	83	65	H1ag1, R1b1a2a1a2c1	11.8
O1	Oakington	Female	420–570 CE	81	50	U5a2a1	3.8
O2	Oakington	Female	385–535 CE	92	68	H1g1	2.7
O3	Oakington	Female	395–540 CE	95	64	T2a1a	8.2
O4	Oakington	Female	400–545 CE	67	77	H1at1	6.3
HS1	Hinxton	Female	666–770 CE	36	91	H2a2b1	4.4
HS2	Hinxton	Female	631–776 CE	42	74	K1a4a1a2b	3.8
HS3	Hinxton	Female	690–881 CE	16	71	H2a2a1	0.9

The ‘% endogenous' values give the percentage of sequenced DNA that map to the human reference genome. The ‘% unique' values give the fraction of mapped reads that are left when excluding duplicates. The ‘mean autosomal coverage' is the number of reads covering a base, averaged across chromosome 20. C14 Dates are calibrated, with 95% confidence intervals given.
