# Supplementary material for: Iron Age and Anglo-Saxon genomes from East England reveal British migration history
Source: Nat Commun. 2016 Jan 19;7:10408. doi: 10.1038/ncomms10408 (PMC4735688; doi:10.1038/ncomms10408)
Supplement: Supplementary Information — Supplementary Figures 1-12, Supplementary Tables 1-4, Supplementary Notes 1-6 and Supplementary References [file ncomms10408-s1.pdf]

Supplementary Figure 1 – Hinxton Site

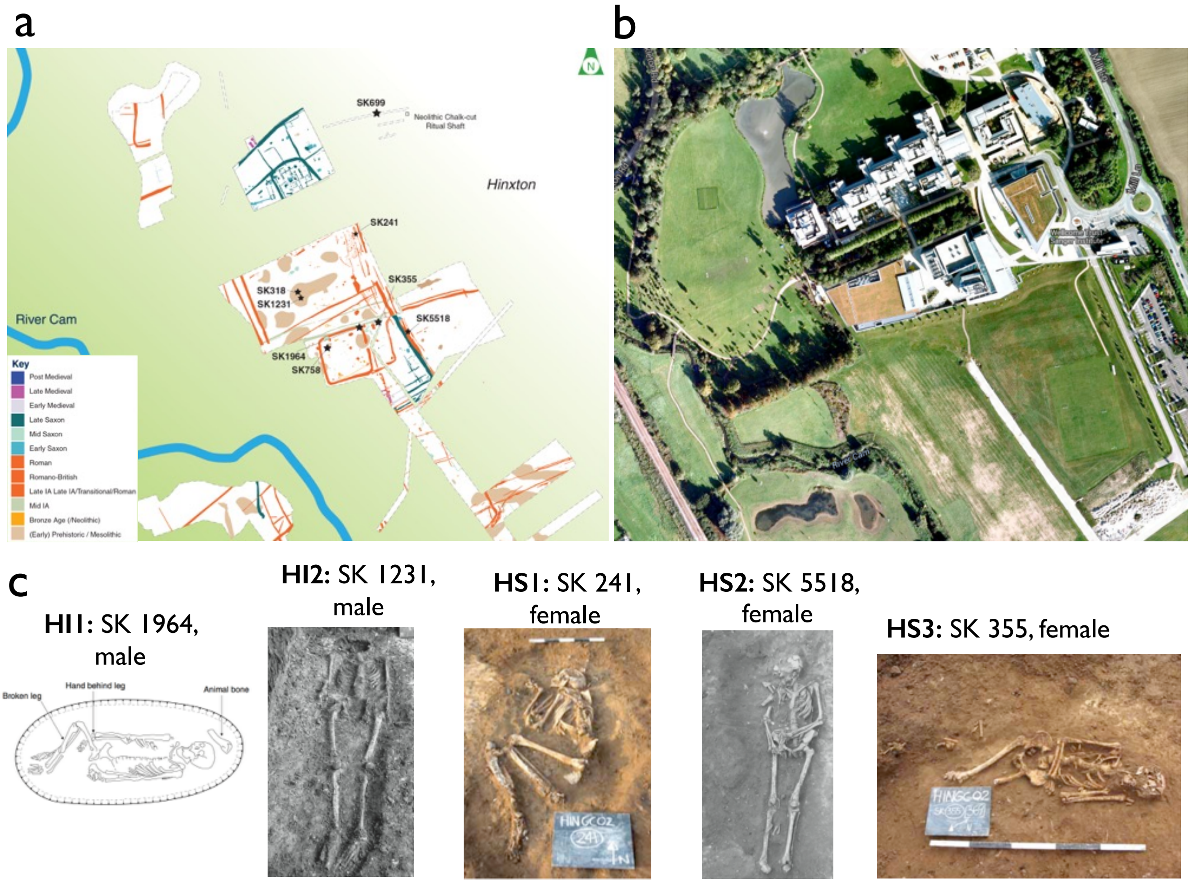

Supplementary Figure 1: Hinxton Site. (a) A plan of the Hinxton archaeological site, with the locations of the skeletal remains. (b) A satellite image of the same area, where today the Wellcome Trust Genome Campus is located. (c) Pictures/Drawing of the 5 samples used in this study.

Supplementary Figure 2 – Oakington Site

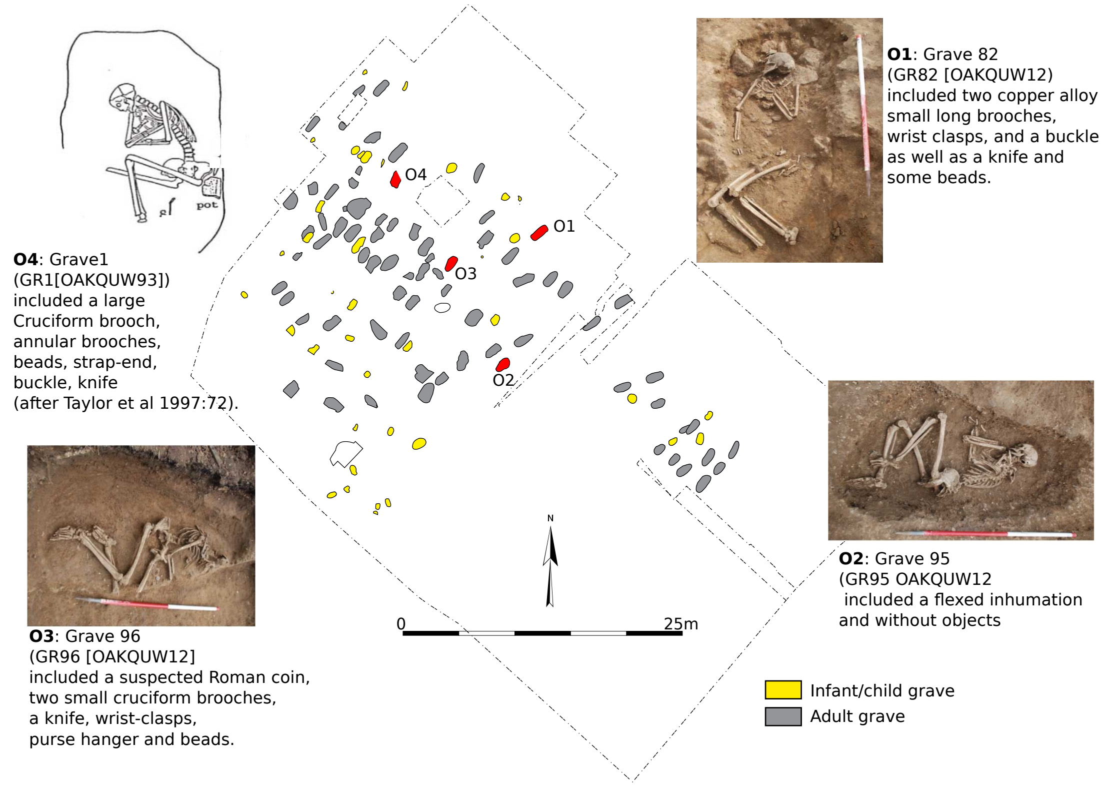

Supplementary Figure 2: Oakington Site. A schematic of the early Anglo-Saxon cemetery in Oakington, with graves colored in grey (adult individuals), yellow (infant individuals) and red (the adult individuals used in this study).

Supplementary Figure 3 - Principal Component Analysis

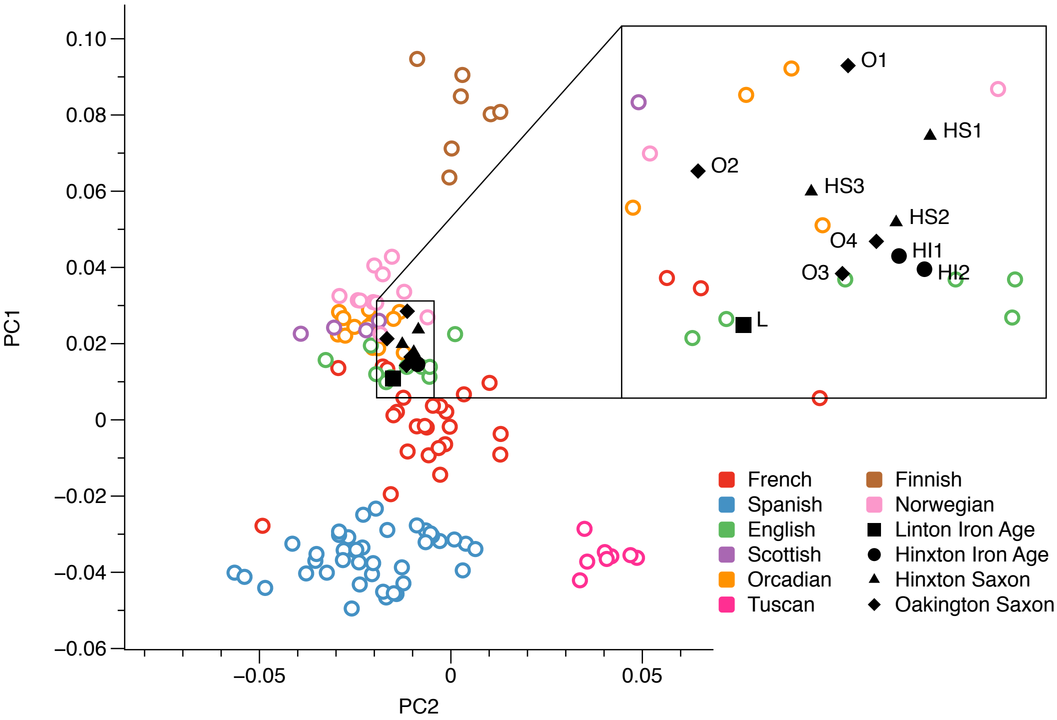

Supplementary Figure 3: Principal component analysis. The first two principal components obtained by analyzing European samples from the Human Origins Data set [10,11] and projecting the ancient samples onto these components. Only populations from Northwestern central Europe are shown. The populations from the Human Origins data set to produce this plot are: Albanian, Bergamo, Bulgarian, Cypriot, Greek, Italian_South, Maltese, Sicilian, Tuscan, English, French, Icelandic, Norwegian, Orcadian, Scottish, Basque, French_South, Spanish, Spanish_North, Belarusian, Croatian, Czech, Estonian, Hungarian, Lithuanian, Ukrainian, Canary_Islanders, Sardinian, Finnish, Mordovian, Russian.

Supplementary Figure 4 – Population Structure in the GBR samples

a)

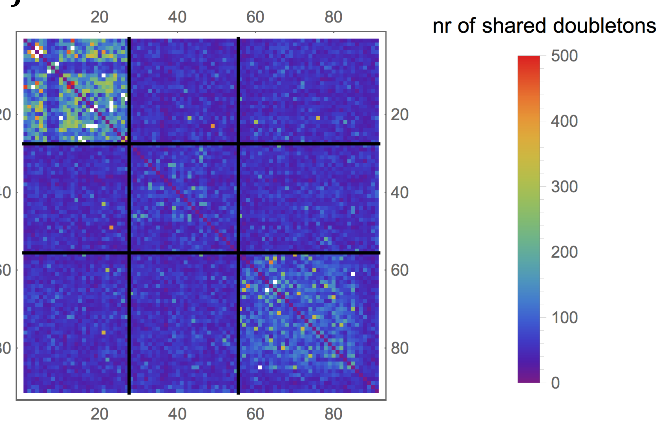

b)

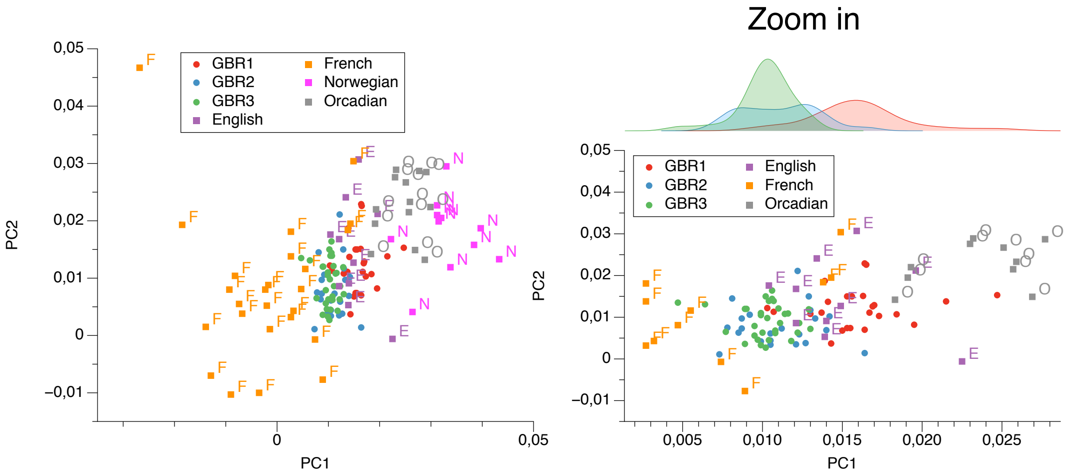

Supplementary Figure 4: Population structure in in the GBR samples. (a) This matrix shows the number of shared doubletons (mutations with allele count 2 within all European 1000 Genomes samples) between two individuals of the 91 GBR samples. The black lines are manually placed to distinguish the three visible clusters. (b) Principal component plot of the 1000 Genomes GBR samples. The three clusters identified in the GBR samples (named GBR1, GBR2 and GBR3) are projected onto selected European samples from the Human Origins data set. We conclude from this analysis that GBR1 corresponds to the Orkney cluster, given its substantially closer location to the Orcadian samples in the PCA plot.

Supplementary Figure 5 – Additional rare variant projections

a)

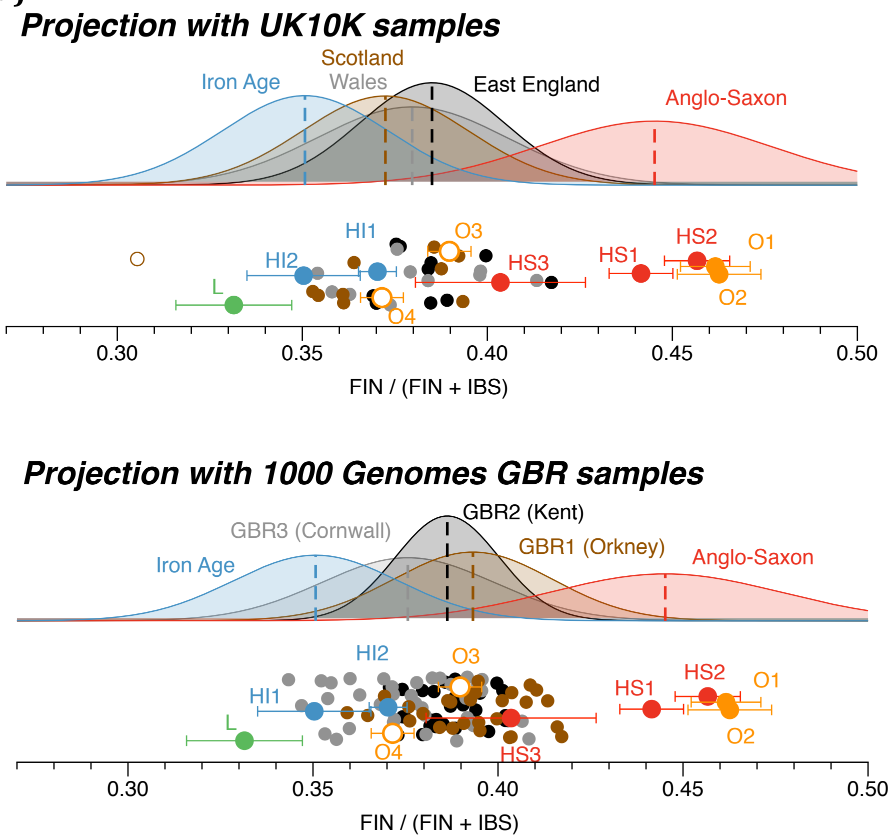

b)

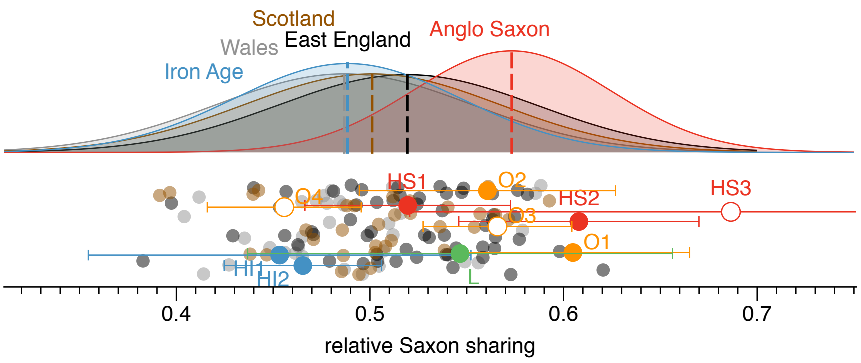

Supplementary Figure 5: Additional rare variant projections. (a) Projection of modern British samples using Finnish vs. Spanish allele sharing, similar to the analysis shown in Figure 2 in the main text and described in Methods, but with the Finnish instead of the Dutch population as an outgroup. The X axes show how many rare variants up to allele count 5 (identified in 433 Europeans) are shared with Finnish samples vs. Spanish samples. The upper plot shows the same modern samples as in Figure 2, from the UK10K project. The lower plot shows 91 modern samples from the GBR population, grouped into three clusters. (b) Allele sharing between UK10K and ancient samples. This figure shows how many rare alleles (identified in 1854 UK10K samples) each UK10K individual from one of the three locations shares with the Anglo-Saxon vs. the Iron Age group (see Supplementary Note 3 for details).

Supplementary Figure 6 – Rarecoal fits of simulated data

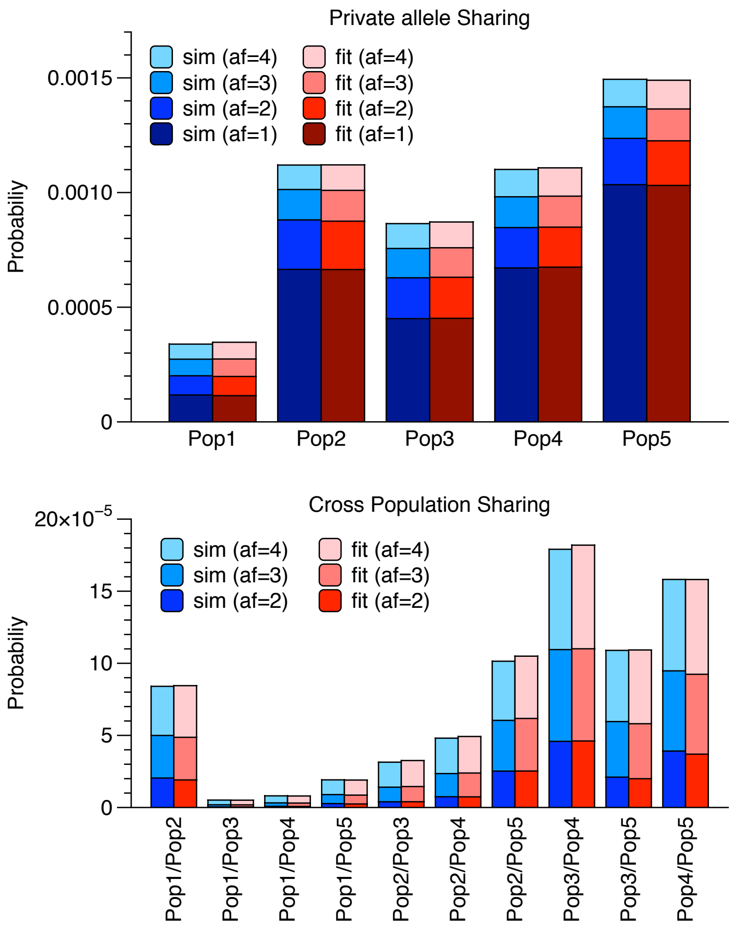

Supplementary Figure 6: Rarecoal fits of simulated data. We compare the theoretical distribution of rare variants predicted by the model estimated in Figure 3b (red) with the true distribution of variants (blue), yielding a good fit of the model given the data. The top panel shows variants private to one population, the lower panel shows variants shared across populations.

Supplementary Figure 7 – Rarecoal estimates under admixture

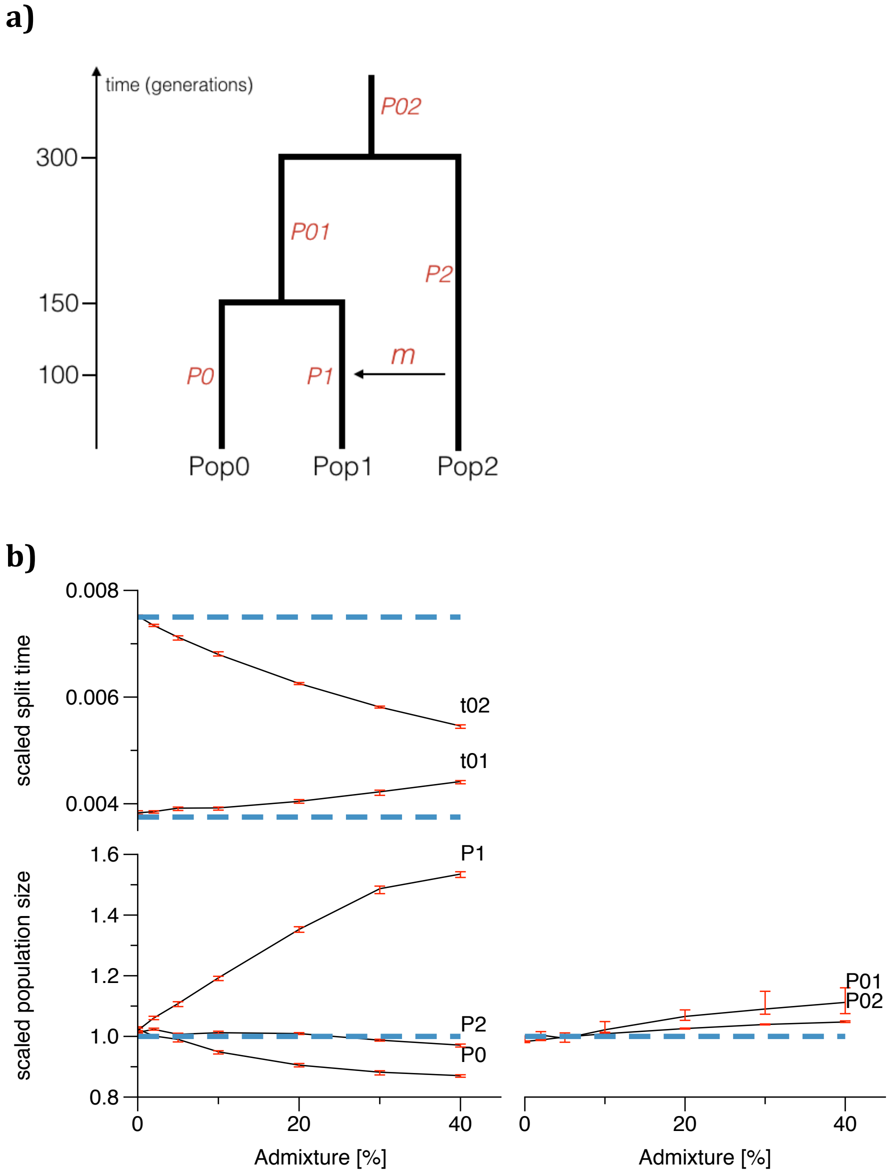

Supplementary Figure 7: Rarecoal estimates of simulations to test robustness under admixture. (a) We simulate three populations, with 100 diploid individuals each, related by two split times, 150 and 300 generations ago. At 100 generations ago, admixture with proportion *m* occurs from Pop2 into Pop1. (b) The dashed blue lines indicate the true value, and the x axis denotes the rate of admixture. As can be seen, increasing admixture leads to an increasing deviation of the estimated split times and population sizes from the true parameters.

Supplementary Figure 8 – Maximum likelihood trees of European populations

a)

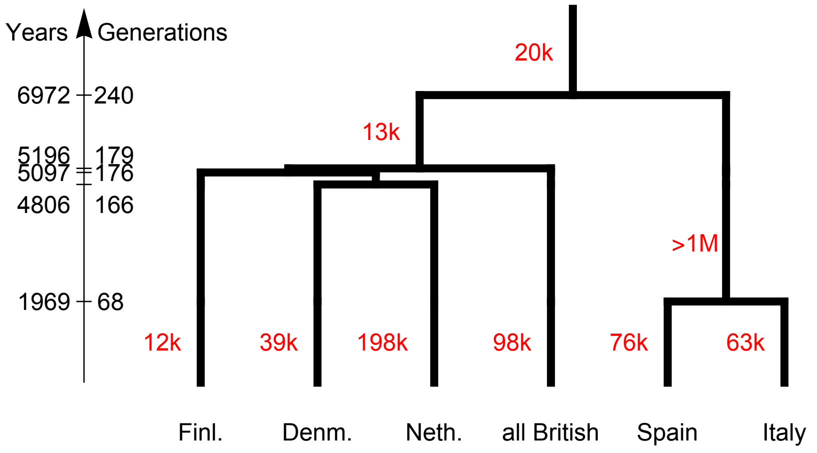

b)

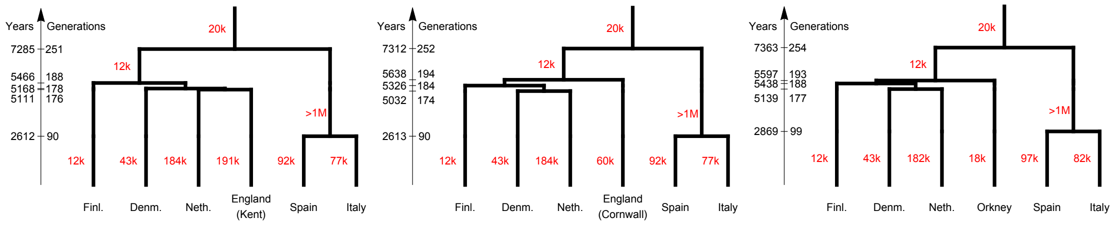

Supplementary Figure 8: Maximum likelihood trees of European populations. (a) European tree estimated from 524 individuals without separating the British samples into subpopulations. Population size estimates are shown in red, split time estimates on the left axis. (b) European trees using the three groups in the GBR sample set separately.

Supplementary Figure 9 – Rarecoal fits of European data

a)

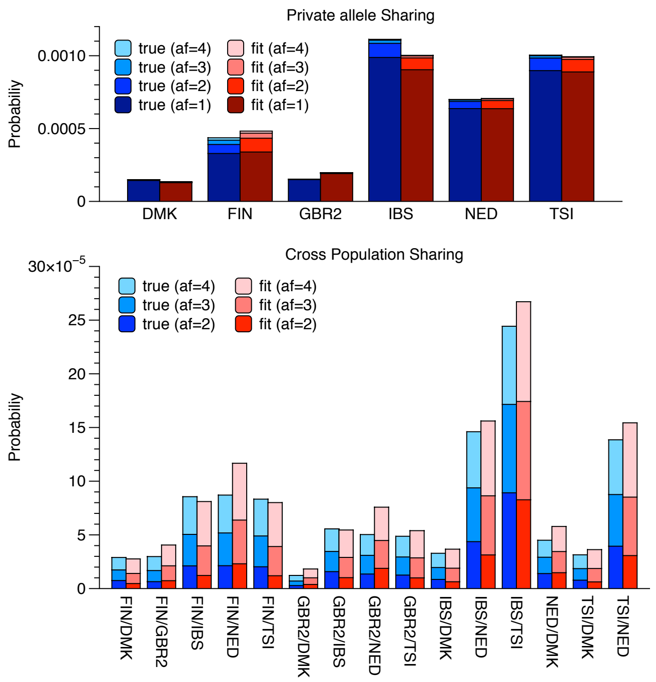

b)

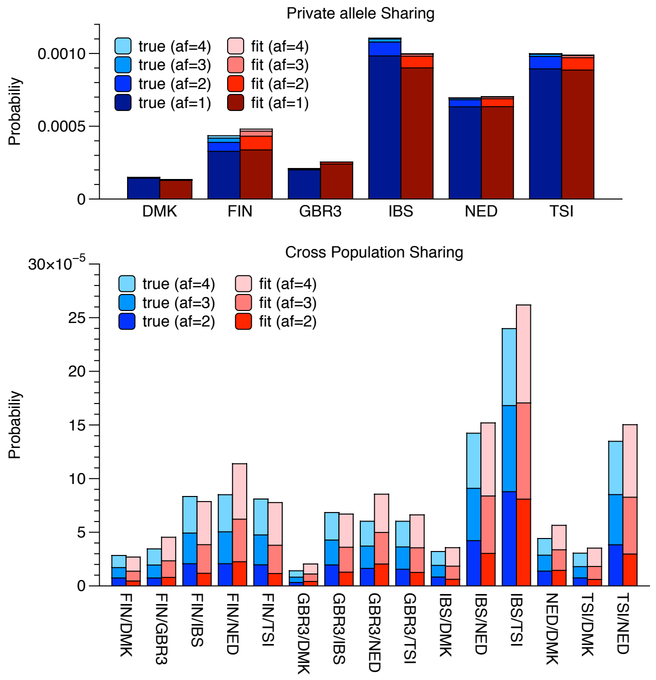

Supplementary Figure 9: Rarecoal fits to European data. Similar to Extended Data Figure 5, we obtain fits between the model obtained on the European samples with the true distribution of rare variants. In a) we fit the tree using samples from Kent (GBR2), as shown in Figure 3c, and in b) we fit the tree shown in Figure 3d, with samples from Cornwall (GBR3). The fit is reasonable, with some systematic differences owing to simplifying assumptions such as constant population sizes and the absence of migration.

Supplementary Figure 10 – Tree mapping using Kent as GBR proxy

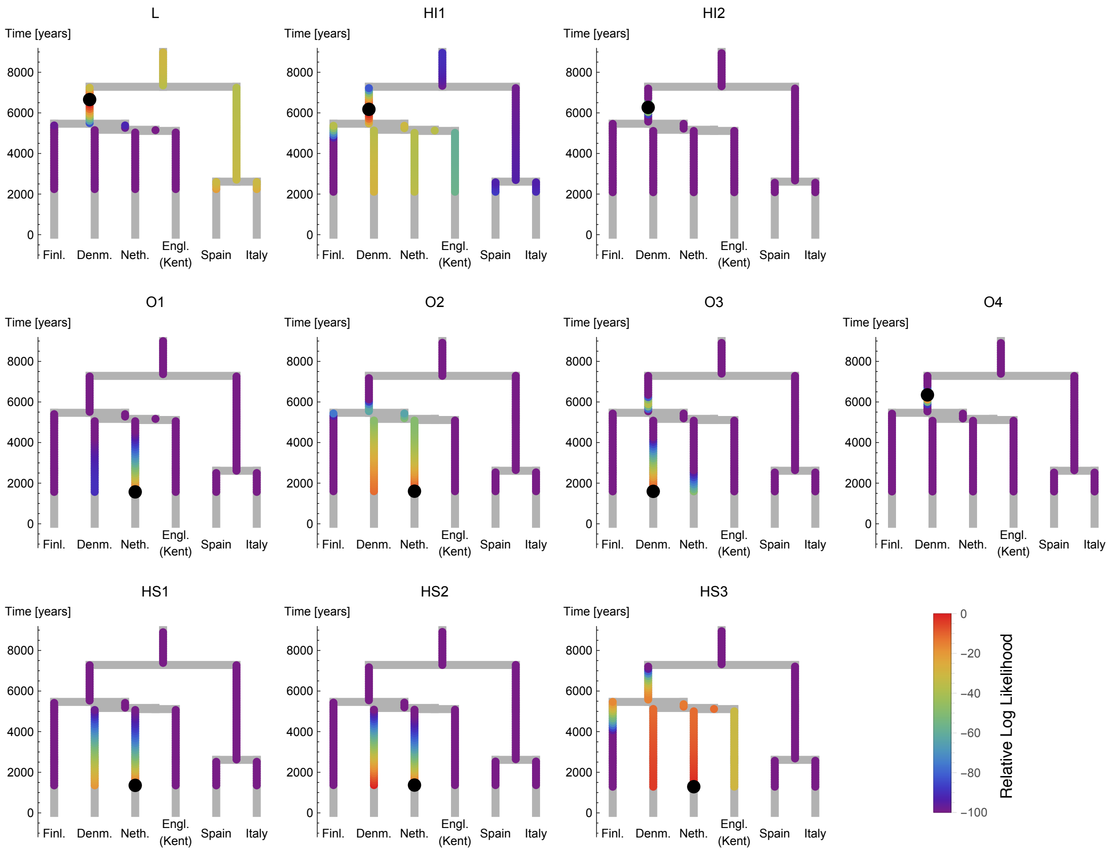

Supplementary Figure 10: Placing ancient samples into the European tree, using the Kent population as British branch. This shows a similar analysis as shown in Figure 4 in the main text (see Supplementary Note 5), but with the Kent population (instead of the Cornwall population) as a proxy for the British branch.

Supplementary Figure 11 – Mapping modern samples onto European tree with Cornwall as British branch

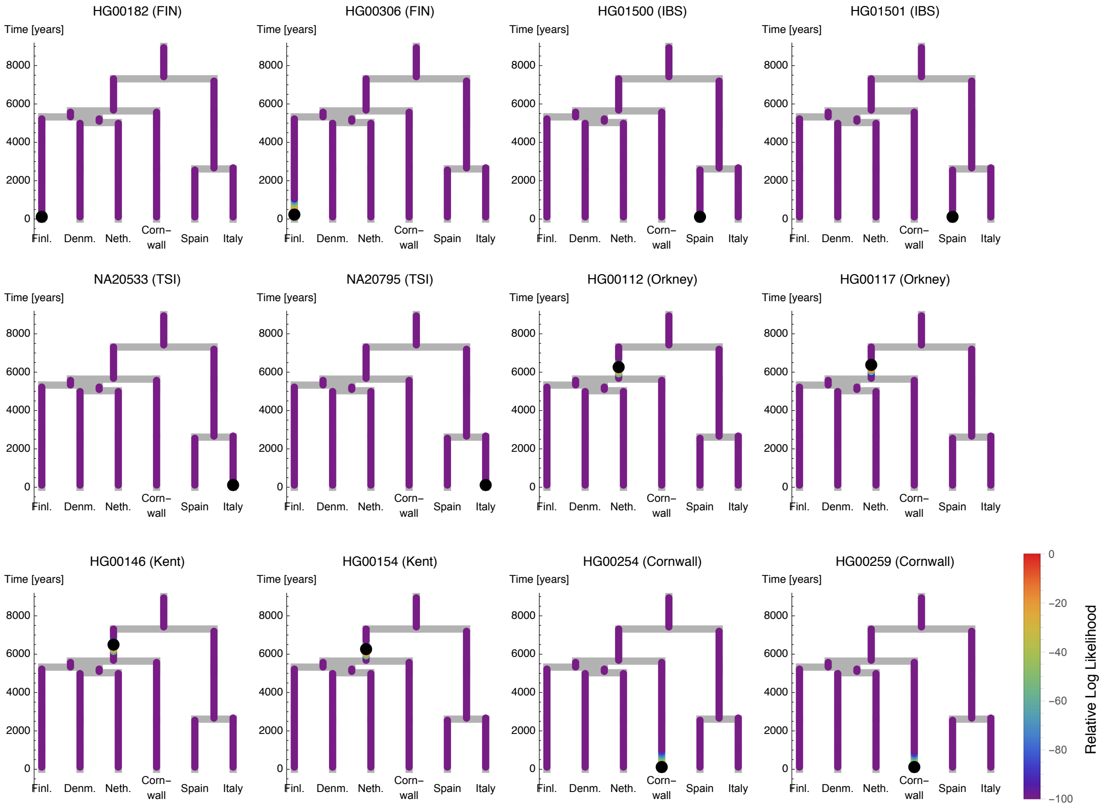

Supplementary Figure 11: Rarecoal tree painting with modern samples. The likelihood surface along the tree (see Supplementary Note 5) for several modern samples from the 1000 Genomes project. Here we used the samples from Cornwall as the proxy for the English population. Most samples map correctly onto the tip of their respective branches, but when we map GBR samples from Kent or Orkney, they map to the Northern European ancestral branch, as expected with an English branch based on Cornwall. The black dot indicates the maximum likelihood merge point onto the tree.

Supplementary Figure 12 - Mapping modern samples onto European tree with Kent as British branch

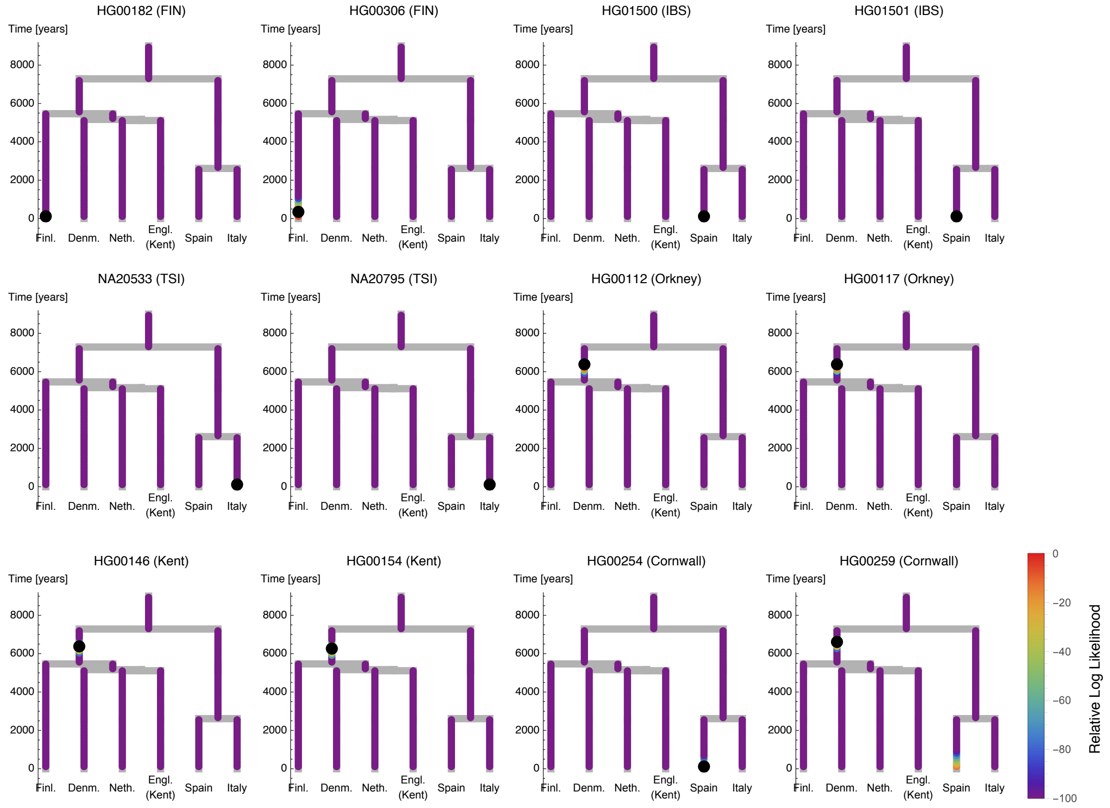

Supplementary Figure 12: Mapping modern samples from 1000 Genomes into a European tree using Kent as British population branch. A similar figure as Supplementary Figure 11, but with Kent used as the British branch, instead of Cornwall.

Supplementary Table 1 – DNA libraries

Library ID	Sample ID	Article ID	Individual/ museum ID	Sample Type	Site	Repair	Complexity	% endog, DNA
**LP26.01**	12880A	HI1	SK1964	Second premolar root	Hinxton	USER	n/a	19%
**LP26.02**	12881A	HS1	SK241	First molar root	Hinxton	USER	n/a	34%
**LP26.03**	12882A		SK758	Lower first molar root	Hinxton	USER	n/a	n/a
**LP26.04**	12883A	HS2	SK5518	Upper right canine root	Hinxton	USER	n/a	39%
**LP26.05**	12884A	HI2	SK1231	Lower third molar root	Hinxton	USER	n/a	85%
**LP26.06**	12885A	HS3	355	Lower second molar root	Hinxton	USER	n/a	19%
**LP49.01**	15548A		Grave 57a (1375)	Upper left 2nd molar	Oakington	UDGhalf	0.5	13%
**LP49.02**	15549A		Grave 59(1395)	Upper left 1st incisor	Oakington	UDGhalf	0.3	55%
**LP49.03**	15550A		Grave 61(1411)	Lower left 3rd molar	Oakington	UDGhalf	0.9	19%
**LP49.04**	15553A		grave 66 (1450)	Lower left 3rd molar	Oakington	UDGhalf	7.9	29%
**LP49.05**	15555A		Grave 78a (1747)	Upper left canine	Oakington	UDGhalf	0.4	41%
**LP49.06**	15556A		Grave 80 (1740)	Lower left 2nd molar	Oakington	UDGhalf	0.1	1%
**LP49.07**	15558A	O1	Grave 82 (1779)	Upper left 2nd molar	Oakington	UDGhalf	9.8	76%
**LP49.08**	15617EBC		extraction blank			UDGhalf		
**LP49.09**	15560A		Grave 85 (1785)	Upper left 1st premolar	Oakington	UDGhalf	0.4	18%
**LP49.10**	15568A		Grave 94 (1866)	Upper right 2nd incisor	Oakington	UDGhalf	0.5	54%
**LP49.11**	15569A	O2	Grave 95 (1870)	Lower right 2nd molar	Oakington	UDGhalf	7.2	89%
**LP49.12**	15570A	O3	Grave 96 (1882)	Lower left canine	Oakington	UDGhalf	26.6	92%
**LP49.13**	15575A		GrAVE 112 (2222)	Lower right canine	Oakington	UDGhalf	0.4	13%
**LP49.14**	15576A		burial 3 (1622)	Lower left 3rd molar	Oakington	UDGhalf	n/a	n/a
**LP49.15**	15577A	O4	burial7 (1633)	Lower left 3rd molar	Oakington	UDGhalf	100	67%
**LP49.16**	15618EBC		extraction blank			UDGhalf		
**LP50.11**	15579A	L	Sk 270	Lower canine	Linton	UDGhalf	4.5	51%
**LP50.12**	15583A		Sk 352	Upper left 2nd incisor	Linton	UDGhalf	0.1	1%
**LP50.13**	15586A		Sk 351	Upper right 3rd molar	Linton	UDGhalf	1.4	12%
**LP50.14**	15589A		Sk 887	Lower right canine	Oakington	UDGhalf	0.3	2%
**LP50.16**	15683EBC		extraction blank			UDGhalf		

Supplementary Table 1: Library preparation details for all samples that were screened. See Methods for details about library preparation. Only those libraries with labels in column 3 were selected for deep sequencing, based on screening results. Values in the “complexity” columns give the fold coverage of the genome expected after hypothetical sequencing of the entirely library.

Supplementary Table 2 – Radiocarbon dates of samples

Sample	Service reference	Uncalibrated conventional age	2-sigma calibrated age
**L**	SUERC-14246	2155±35BP	360 - 50 BCE
**HI1**	OxA-29573	2039 ±27	160 BCE - 26 CE
**HI2**	Wk-12599	2029±49BP	170 BCE - 80 CE
**O1**	Beta-397731	1560±30 BP	420 - 570 CE
**O2**	Beta-397732	1620±30 BP	385 - 535 CE
**O3**	Beta-397733	1600±30 BP	395 - 540 CE
**O4**	Beta-397734	1590±30 BP	400 - 545 CE
**HS1**	OxA-29573	1288 ±25	666 - 770 CE
**HS2**	OxA-X-2565-12	1320± 45	631 - 776 CE
**HS3**	OxA-29572	1230 ±25	690 - 881 CE

Supplementary Table 2: Radiocarbon dates of samples. The table gives the uncalibrated and calibrated C14-dates for all sequenced samples. The reference starting with SUERC is from the *Scottish Universities Environmental Research Centre.* The reference starting with Wk is from the *University of Waikato Radiocarbon Dating Laboratory.* The references starting with OxA are from the *Oxford Radiocarbon Accelerator Unit.* The references starting with Beta are from *Beta Analytic Radiocarbon Dating.* Calibrated dates are computed from the Oxcal computer program (v4.2) of C. Bronk Ramsey, using the 'IntCal13' dataset.

Supplementary Table 3 – Contamination estimates

Sample	mtDNA Coverage	Informative Sites	N_{cons}	N_{alt}	mtDNA estimate	Nuclear estimate
**L**	78	0			n/a	0.00012
**HI1**	1145	4	5341	3	0.00056	0.00005
**HI2**	2177	1	2473	13	0.0052	0.00887
**O1**	642	3	9168	0	<0.00033	0.01495
**O2**	652	0			n/a	0.01219
**O3**	410	6	35913	4	0.00011	0.01312
**O4**	255	0			n/a	0.01505
**HS1**	1020	4	4197	3	0.00071	0.01090
**HS2**	537	7 (6)	3290 (2673)	89 (10)	0.027 (0.0037)	0.01018
**HS3**	587	6	4206	0	<0.00071	0.00009

Supplementary Table 3: **Contamination estimates**. DNA contamination estimates based on mitochondrial and nuclear DNA. Numbers are contamination fractions on a 0-1 scale. For O2, O4 and L, no mtDNA estimate could be generated because there were no informative sites. The relatively high contamination estimate of HS2 is due to a single site in the hypervariable region, which could reflect natural heteroplasmy. The estimate without that site is given in parentheses for that individual. See Supplementary Note 2 for details.

Supplementary Table 4 – Estimates of the Anglo-Saxon ancestry fraction in modern Britain

Data set	Group	With HS3	Outgroups	Anglo-Saxons	Anglo-Saxons StdDev	Iron Age	Iron Age StdDev	Value for Group	StdDev for Group	Fraction	StdDev
**UK10K**	East	Yes	Dutch,Spanish	0,607	0,015	0,504	0,026	0,543	0,013	**0,38**	**0,21**
**UK10K**	Wales	Yes	Dutch,Spanish	0,607	0,015	0,504	0,026	0,535	0,013	0,30	0,22
**UK10K**	Scotland	Yes	Dutch,Spanish	0,607	0,015	0,504	0,026	0,536	0,013	0,31	0,22
**UK10K**	East	No	Dutch,Spanish	0,614	0,007	0,504	0,026	0,543	0,013	0,35	0,19
**UK10K**	Wales	No	Dutch,Spanish	0,614	0,007	0,504	0,026	0,535	0,013	0,28	0,21
**UK10K**	Scotland	No	Dutch,Spanish	0,614	0,007	0,504	0,026	0,536	0,013	0,29	0,21
**UK10K**	East	Yes	Finnish,Spanish	0,445	0,02	0,351	0,016	0,385	0,014	**0,36**	**0,20**
**UK10K**	Wales	Yes	Finnish,Spanish	0,445	0,02	0,351	0,016	0,380	0,018	0,31	0,23
**UK10K**	Scotland	Yes	Finnish,Spanish	0,445	0,02	0,351	0,016	0,372	0,016	0,22	0,22
**1000G**	Kent	Yes	Finnish,Spanish	0,445	0,02	0,351	0,016	0,386	0,01	**0,37**	**0,17**
**1000G**	Cornwall	Yes	Finnish,Spanish	0,445	0,02	0,351	0,016	0,376	0,016	0,27	0,22
**1000G**	Orkney	Yes	Finnish,Spanish	0,445	0,02	0,351	0,016	0,393	0,015	0,45	0,21
**UK10K**	East	No	Finnish,Spanish	0,456	0,008	0,351	0,016	0,385	0,014	0,32	0,17
**UK10K**	Wales	No	Finnish,Spanish	0,456	0,008	0,351	0,016	0,380	0,018	0,28	0,20
**UK10K**	Scotland	No	Finnish,Spanish	0,456	0,008	0,351	0,016	0,372	0,016	0,20	0,20
**1000G**	Kent	No	Finnish,Spanish	0,456	0,008	0,351	0,016	0,386	0,01	0,33	0,14
**1000G**	Cornwall	No	Finnish,Spanish	0,456	0,008	0,351	0,016	0,376	0,016	0,24	0,19
**1000G**	Orkney	No	Finnish,Spanish	0,456	0,008	0,351	0,016	0,393	0,015	0,40	0,17

Supplementary Table 4: Estimates of the Anglo-Saxon ancestry fraction in modern Britain. Estimates of the Anglo-Saxon component in the modern British population, using different outgroup populations (Dutch and Finnish vs. Spanish) and different British populations as test cases. We include both the case with and without HS3 as a member of the Anglo-Saxon group. O3 and O4 are always excluded because they seem admixed or of non-Anglo-Saxon ancestry (see Figure 2 in the main text). The three estimates including HS3 for the East of England or Kent are highlighted. Details on how the values in this list are computed can be found in Supplementary Note 3.

Supplementary Note 1 – Archaeological sites and sample descriptions

Linton Site

Between 2004 and 2010 investigations by Oxford Archaeology East (funded by Cambridgeshire County Council) on land at Linton Village College, Cambridgeshire (NGR TL 55547 46984), produced evidence of over four and a half thousand years of human activity. The *c.*8ha site lies in an agriculturally rich area on the lower valley slopes of the River Granta, just outside the village of Linton. A range of features and deposits of later Neolithic to post-medieval date was revealed across most of the areas investigated. These included a series of later Neolithic Grooved ware pits, two ring-ditches (remains of burial mounds), a Middle to Late Bronze Age enclosure and later Iron Age settlement evidence; the latter associated with an inhumation and metalworking debris of the same date. Roman features included a field system and trackway, in addition to the remains of a possible animal-powered mill and a number of neonate burials. Post-Roman activity was represented by an Early Saxon enclosure, five Middle Saxon inhumations (a possible execution cemetery) and a quantity of 17th-century items possibly related to a documented Civil War skirmish.

Analysed sample from Linton

<u>Linton Skeleton 270 (AKA 2270) (sample L in the main Text):</u>

A poorly-preserved contracted ('crouched') inhumation of a female aged over 50 in a shallow, oval grave (1.1m x 0.7m) located in proximity to an area of settlement-related features. The burial was aligned north to south, and the skeleton was laid on its right side, with the head facing west. Analysis of the skeleton revealed that the individual was 1.58m (+/- 4.3 cm) tall. Osteoarthritis and spondylosis deformans were present in her spine and wrist, while enamel hypoplasia indicates that she experienced health stress during childhood.

Additional samples (Anglo-Saxon) from Linton

<u>Linton Skeletons 351 and 352</u>:

A group of three graves containing five skeletons was uncovered in the area of a former Roman trackway. One of the graves, aligned north-east to south-west, contained three individuals (sks 350, 351 and 352) that were all apparently buried during a single event. The grave was sub-rectangular, with steeply sloping sides and a flat base, and measured 1.91m long, 0.92m wide and 0.20m deep.

The initial burial appears to have been that of an older child of around 12 years of age (sk 352), who had been positioned along the eastern side of the grave in a supine position with the head to the south-west. Some pathological changes were noted on this skeleton including evidence for growth arrest, metabolic disease (cribra orbitalia and porotic hyperostosis) and mild trauma. No evidence for peri-mortem injuries was observed. This burial was followed by the interment of a child of around five years of age (sk 350) that was placed in the south-west corner of the grave.

The final burial was that of a mature adult female, aged over 45 (sk 351), who had been placed centrally in the grave on top of skeletons 352 and 350. This

individual had been decapitated prior to burial and the head had been deposited within the grave first. The skeleton was in a loosely extended, supine position with the feet to the south and right femur lying over the top of the skull. Both arms were flexed at the elbows, with the left arm lying across the torso and the right angled outwards 'akimbo' from the body. Several pathological conditions were observed, including developmental anomalies, maxillary sinusitis, Schmorl's nodes and joint disease. Peri-mortem sharp-force trauma, associated with head removal, was present on the fourth and fifth cervical vertebrae.

Hinxton Site

Extensive archaeological investigations were undertaken in Hinxton, South Cambridgeshire by Oxford Archaeology East between 1993 and 2014 on behalf of the Wellcome Trust [1,2]. The investigations, which centred around Hinxton Hall and the Genome Campus, extended on either side of the River Cam and were set within a rich archaeological landscape (Supplementary Figure 1). The ancient course of the Icknield Way crosses the site, which itself lies 1.5 kilometres north of the Roman town at Great Chesterford. This post-glacial valley landscape attracted humans to hunt and make flint tools from the Late Upper Palaeolithic (*c*. 10,000 BC) and into the Mesolithic and Early Neolithic periods until eventually the first tree clearances to enable farming and more permanent settlement began. This area also became a focus for more ceremonial activities associated with the dead during both the Middle Bronze Age and the Iron Age to Roman periods, represented by burials and a mortuary enclosure. From the Middle Iron Age until the Middle Romano-British period the site appears to have been in continuous agrarian use, specialising in animal husbandry, until its apparent abandonment.

The land was not resettled until the Early to Middle Saxon period when activity included a small scatter of timber houses and sunken-featured buildings and associated features. By the Late Saxon period, settlement had coalesced in the northern part of the site (Hinxton Hall), associated with an ordered field system. During the 11th century a large ditch enclosed the settlement, and several new timber buildings were constructed. This may have been the documented Hengest's Farm, which gave modern Hinxton its name. Further Late Saxon discoveries were made in Ickleton, on the western side of the River Cam, where a working area probably associated with flax retting and wood working was found. To the south of the main enclosed settlement were the remains of a small hamlet, also occupied during the Saxo-Norman and earlier medieval period and seemingly abandoned by the early 13th century. A number of Anglo-Saxon burials were scattered around the eastern limits of the settlements, buried within silted up ditches and pools and within an isolated grave.

Analysed samples from Hinxton

<u>Skeleton 1964, sample HI1 in the main text:</u>

Skeleton 1964 was that of an old male, buried supine with its legs extended, within a grave located in the north-east corner of the mortuary enclosure. Analysis indicates that this skeleton was dolichocranic, or had a relatively long skull, and had maxillary sinusitis, vertebral disc herniation (Schmorl's nodes) and an oblique fracture of the right lower leg that had healed. At 159.0 cm tall, the individual was within the normal range for the period. Dental pathology was

observed indicating that the individual had periodontal disease, advanced caries, abscesses and had also lost all of their molars and lower right second premolar before death.

Skeleton 1231 (sample HI2 in the main text):

An isolated burial placed within an infilled pond that had also previously contained a Bronze Age skeleton. The Late Iron Age/Early Roman skeleton was that of a middle/old adult male who had been placed in a north-east to south-west orientated grave in an extended, supine position with their arms by their side and their head in the north-east. Their stature was 174.1cm. They had lost a number of teeth prior to death and the skeleton also displayed evidence of caries and abscesses. In addition to showing evidence of joint disease (osteoarthritis), Schmorl's nodes, maxillary sinusitis and metabolic disease (cribra orbitalia), some pathological changes were observed may have been caused by repetitive activity involving the shoulder from a young age.

Skeleton 241, sample HS1 in the main text:

Buried within a shallow oval grave cut into the top of a major boundary ditch, skeleton 241 was that of a middle aged/old female placed in a crouched position. This individual measured 158.6 cm in stature. Ante mortem tooth loss had affected the two lower mesial incisors only, and this unusual position may indicate that an occupational use of the teeth, or perhaps trauma, had resulted in their loss. Other dental conditions included caries and periodontitis. Osteoarthritis was present on some joints, while evidence of Schmorl's nodes and metabolic disease (cribra orbitalia) was also observed.

Skeleton 5518, sample HS2 in the main text:
A very large sub-oval grave or pit lay to the south of that containing sk 241, and was also cut into the boundary ditch: it contained the skeleton of a middle aged/old female (50+) that was in a supine position. This individual measured 153.6 cm in stature and had suffered ante mortem tooth loss, caries and abscesses; evidence of trauma, Schmorl's nodes non-specific bone inflammation and joint (including osteoarthritis) and metabolic disease were also present.

Skeleton 355, sample HS3 in the main text:
A grave located adjacent to the entrance way of an enclosure contained the skeleton of a young/middle adult female. Buried in a supine position with her legs flexed, the skeleton was aligned roughly north to south with the arms lying across the abdomen. This individual had an estimated stature of 163.5 cm and showed evidence of Schmorl's nodes and trauma, including a healed fracture on the right arm.

Additional samples from Hinxton
Skeleton 758 (Middle to Late Iron Age)

Skeleton 758 was an adolescent (less than 16 years) of unknown sex buried within the north-east corner of the mortuary enclosure, where it had been inserted into the top of an existing pit. The individual was buried supine with the legs extended and arms by their sides. Schmorl's nodes were present on the spine.

Oakington Site

Early Anglo-Saxon Cemeteries

Furnished Anglo-Saxon burials have been studied for nearly three centuries, based on radiocarbon dates and artistic styles we know that these equipped graves date between the late fifth and early eighth centuries [3]. The earliest phase of burial rituals dates to the fifth and sixth centuries and have been referred to as Migration Period, Pagan or early Anglo-Saxon graves [4]. These cemeteries are predominantly found in the south and east of England from Dorset to Northumberland with regional variation evident within the burial rite [5]. Grave goods include weapons, for example; spears, swords or shield bosses. Grave goods might also be dress objects, for example; brooches, beads, pins or buckles. Also included are containers, parts of animals or Roman artefacts curated and deposited hundreds of years after their manufacture, for example; spoons, coins or rings and brooches. Grave furnishings like these vary according to male or female gender and with age [6]. Many graves have no surviving artefacts at all, and we can only speculate about the organic furnishings which may have been present.

In the early 20th century archaeological interpretations attributed these graves to specific Historical narratives, for example, Anglo-Saxon migration or invasion events. More recent interpretations, however, do not consider funerals to have been the product of static cultural processes, but dynamic and mutable interactions during which communities and individuals expressed and constructed their own identities [7-9]. Participants at these events were associates with different backgrounds including, but not limited to; extended families, households, kinship groups, dependents (slaves and/or children) and social elites depending on who the deceased was. Each burial event was unique and each one was specific to and contingent upon a particular historical moment meaningful to the community that created it.

Oakington early Anglo-Saxon Cemetery

Oakington is a small village in Cambridgeshire, UK, seven kilometres northwest of Cambridge. It was named *Hochinton* and *Hochintone* in the Domesday Book of AD 1086 (VCH 1989:192-195). The Oakington early Anglo-Saxon cemetery was first identified in 1926 when three burials were found as a result of cultivation [10]. The site (Supplementary Figure 2) was rediscovered in 1993 during the construction of a children's playground and in 1994 the Cambridge County Council's Archaeological Field Unit excavated an area of 140 sq. m, identifying 24 human skeletons [11]. In 2000 the 1993-94 skeletons were interred within a brick lined vault to the west of the excavated area. In 2006 and 2007 the same archaeological group, then known as CAMARC, excavated a further area of 450 sq. m ahead of the construction of the village's new Recreation Centre, the excavators recorded 17 skeletons. Between 2010 and 2015 the cemetery was systematically excavated by a University of Central Lancashire team (UCLan), with support from Oxford Archaeology East (OAE, formerly CAMARC) and with outreach activities organised by members of staff from Manchester Metropolitan University [12].

By the end of the final excavation season in 2014, a total of 128 individuals had been excavated from an area of approximately 1800 sq. m. Radiocarbon dates

from the skeletal remains and the artefacts from within the graves provide a primarily sixth century date for the cemetery. Preliminary skeletal investigations show that 34 individuals were female, 25 male, 7 adults remain unidentified, 27 individuals were sub-adults aged between 6 and 12, and 35 were below the age of 5. This unusually high number of younger individuals may identify Oakington as a central place in a regional kinship network [13]. The artefacts from the 2010-2014 excavations are currently being conserved and the skeletal remains are being analysed for publication.

Samples used in this study

Oakington [OAKQUW93/11] 1633 Grave 1 (O4 in the main text) was the first grave excavated in 1993 during the playground development, she was a female in her 'mid 40s' and was 1.61m or 5'3" tall [11]. The body was positioned on her right hand side with the head to the south west of the grave facing down towards the knees. She was buried facing east and positioned with her legs flexed forward and arms crossed at her chest. The grave was furnished with a large cruciform brooch, a pair of wrist clasps, a pair of annular brooches, 14 amber beads, two blue beads, a silver coloured glass bead and a large pot sherd. She was also found with a strap-end, knife and a D shaped iron buckle. In 2000 the skeleton was buried in a vault adjacent to the cemetery site. This vault was excavated by the UCLan team in 2012 and the 1633 remains were found stored within labelled containers.

Oakington [OAKQUW12] 1779 (O1 in the main text) was in grave 82 and was excavated in 2012 by the UCLan team. The grave contained the remains of an adult female laid with her head to the south of the grave and facing east. She was positioned on her back with legs slightly flexed to the right. Her left arm crossed over the torso and was placed over the right chest area. The grave was furnished with two copper alloy small long brooches, a pair of wrist clasps, a buckle, a knife and some beads. Preservation within this grave is mixed, the skull is in good condition but the lower part of the body and pelvis was missing, probably as a result of burrowing.

Oakington [OAKQUW12] 1870 (O2 in the main text) was in grave 95 and was excavated in 2012 by the UCLan team. The grave contained the remains of an adult female laid with her head to the south and facing east. She was positioned on her right hand side with legs flexed forward and crossed. Her arms were placed out in front and her left arm was flexed at the elbow to position her hand under her chin. This grave was not furnished with objects.

Oakington [OAKQUW12] 1882 (O3 in the main text) was in grave 96 and excavated in 2012 by the UCLan team. An adult female laid with her head to the south and facing west. The body was placed on the left hand side with legs crossed and slightly flexed, her arms and hands were positioned to the front. The grave was furnished and included two small copper alloy cruciform brooches, a knife, wrist-clasps, purse hanger, two beads and a perforated copper disc, which may have been a Roman coin. The skeleton was truncated by the construction of the playground and was missing parts of the right tibia and fibula, sections of both radius and ulna and a portion of the skull.

Other Graves Sampled

Oakington Sk887 [OAKQUW07] grave 40. An adult female buried supine with her head to the south. Her left leg was flexed placing her foot under the right leg below the knee. Her right arm was flexed and her hand was placed on the abdomen area. The grave was furnished with 77 amber and glass beads, a pair of wrist clasps, two small copper alloy cruciform brooch, a Roman finger ring, an iron buckle and an iron knife.

Oakington [OAKQUW11] 1375 grave 57a. An adult female aged between 25 and 30 years, she was buried supine with her lower left arm flexed to place her hand over the abdomen area. The grave was furnished with a cruciform brooch and two small long brooches, 21 amber beads, 4 glass beads, wrist clasps, belt fittings and an iron knife. The woman in grave 57 had a foetus across her pelvic cavity, this foetus lay low and transverse suggesting an obstetric problem such as shoulder presentation, and was probably the cause of this double fatality [4].

Oakington [OAKQUW11] 1395 grave 59. An adult female buried flexed on her right side with her head to the south and facing east. Her arms were placed in front of her and crossed over, her left arm was placed on the left knee. This grave was furnished with two copper alloy small long brooches, glass beads and wrist clasps.

Oakington [OAKQUW11] 1411 grave 61. An adult female buried supine with her head to the south and facing east, it appears to be slumped forward over her chest. She was buried with two decorated gilt saucer brooches of a Cambridgeshire type, wrist clasps, an iron knife and an iron purse ring.

Oakington [OAKQUW11] 1450 grave 66. An adult female buried supine with her legs crossed and her lower right arm placed over the stomach area. Her head was to the south and faced west. She was buried with a complete pottery vessel to the south of the grave placed by the head. She had a number of amber beads and two pierced copper alloy pendants. She was also buried with two trefoil small long brooches, a pair of wrist clasps, a copper alloy pin, and iron key/latch lifter belt hanging set and a Roman spoon. She had a large pottery fragment at her feet.

Oakington 1740 [OAKQUW11] grave 80. An adult female buried in a semi flexed position on her right hand side head to the south and facing east. Her right elbow was placed in front, and her hand reached back to clasp a set of beads at her chest. Her left arm was flexed at the elbow. Her lower legs were truncated by the 1993/4 excavation. The grave was furnished with 46 amber beads and 22 glass beads in at least two strings, she had two small silvered disc brooches, strap end, wrist clasps, and an iron girdle hanger which included an iron ring, latch lifters and a copper alloy chatelaine. She was also found buried with a fully articulated bovine.

Oakington [OAKQUW12] 1866 grave 94. An adult male [?] buried supine with the head to the south and slumped onto the chest. His left arm was flexed at the elbow and his hand was placed over his chest. His right leg was flexed over the left at the knee crossing the right leg twice. The grave was furnished with a knife.

Oakington [OAKQUW12] 1785 grave 85. An adult female, buried in a flexed position to the left with her head to the south. Her right arm was placed over the

abdomen. The grave was furnished with a bone comb, an iron ring and an iron knife.

Oakington [OAKQUW12] 1747 grave 78a. An adult female buried in a double grave alongside a child. The adult was buried prone with the head to the south and face down. Her legs were crossed and may have been tied. Her right arm passes under her body and the right hand was positioned to clasp a collection of beads and a brooch by the left side of the head. Her left arm passes under her body and her fingers were resting on the child's left arm. The adult was furnished with 17 glass beads, wrist clasps, a small long brooch, an iron knife and an animal bone.

Oakington [OAKQUW13] 2222 grave 112. An adult [?] skeleton buried supine with the head to the south and facing east. The spine curved to the east and both arms were slightly flexed with both hands over the pelvis. The grave was furnished with a knife between the hands and the pelvis.

Acknowledgments for Oakington excavations

Dr Ran Boytner, the Institute of Field Research and the Heritage Lottery Fund for supporting the project. Dr Allison Jones and Dr Gary Bond (UCLan) for funding and administrative support. Dr Faye Sayer and Alison Draper (Manchester Metropolitan University) for outreach and conservation work. Dr Ash Lenton (Australia National University) for on-site surveying. For supervision work during the excavations we would like to thank: Dr Rob Wiseman (Oxford Archaeology East), Sam D Dickinson, Allison Card, Rick Sayer, Meredith Carroll, Vicki Le Quelenec, Tracy Shuttleworth, Kie Leeming, Clare Bedford, Caitlin Halton, Alex Batey, James Hodgsen, Debbie Sale and Justine Biddle.

Supplementary Note 2 - Mitochondrial DNA and Y chromosome analysis

The haplotyping was done by calling consensus sequences using samtools 0.1.19 and bcftools version 1.1, with "samtools mpileup -u -t DPR -r MT", and "bcftools view -v snps". This lists snps that differ from the reference rCRS, which belongs to haplogroup H2a2a1. The haplogrouping was handcurated using the phylotree build 16 from www.phylotree.org [14]. There were a few private snps, as is to be expected from ancient samples, see the table below. We also note that the sample HS3 was a perfect match with the rCRS, apart from one indel.

The haplogroups (listed the following table) are among the most common modern haplogroups in the UK. The haplogroup H1 is found in 13,83% of modern 1000 genomes GBR samples, H in 20,21%, T in 11.43%, K1 in 1.06% and U5 13,83%, [15]. Approximate times for haplogroups can be inferred from [16], and based on these, the age of the haplogroups of our samples are between 8,501 years and 1,428 years with large error margins. The ages of each individual haplogroup is consistent with the radiocarbon dating of the samples.

Individual	MT Haplogroup	Private SNP positions	Age of haplogroup [years]
**HI1**	K1a1b1b	195	4471
**HS1**	H2a2b1	72, 195	2088
**HS2**	K1a4a1a2b		2276
**HI2**	H1ag1	152	2312
**HS3**	H2a2a1		2094
**O1**	U5a2a1	150	2636
**O2**	H1g1		1901
**O3**	T2a1a	7941	2165
**O4**	H1at1		2935
**L**	H1e	14110, 16362	2026

Several previous studies have associated the haplogroup U5 with hunter-gatherer origins, and the haplogroups H,T, K1 as having Neolithic origins in Europe, see [17] and references therein.

Y chromosome haplogroups

The Y haplogroups were called by first calling Y chromosome genotypes using "samtools mpileup -u -r Y -f ". The coverage of the HI1 sample was very low on the Y chromosome, and therefore we restricted our attention to the unique regions within the male-specific part of the Y chromosome reference sequence, that spanned 8.97 Mb in nine separate regions[18]. The Supplementary Table S1 in Wei, et al. [18] was used to filter our Y chromosome calls in HI1 and HI2. We did not do any further filtering, in the hope of capturing at least a few diagnostic SNPs. We compared the informative SNPs to the ISOGG database (http://www.isogg.org/tree/), and determined that the haplogroup of HI1 is R1b1a2a1a2c, and the haplogroup of HI2 is R1b1a2a1a2c1.

The coverage on HI1 on the diagnostic sites is 1x up to 3x, using a minimum mapping quality of 37. We have 7 derived alleles and 7 ancestral alleles.
If we exclude the sites where the allelic state is T or A in the transition polymorphism, we have two markers (L21, S461) left supporting the haplogroup R1b1a2a1a2c, so we conclude that HI1 was probably in haplogroup R1b.

For HI2, the coverage ranges from 1x to 14x on the diagnostic sites is with the mapping quality of 19 and above. We have 15 ancestral alleles and 13 derived alleles. If we exclude the sites where the allelic state is T or A in the transition polymorphism and require mapping quality of at least 30, we have markers D1857, P241, CTS3575, L21, S245, S461. These markers point to the haplogroup R1b1a2a1a2c. It is therefore possible that both HI1 and HI2 could be in the same haplogroup. HI2 has the marker M269, while there is no coverage on HI1 on that site. The incidence of haplogroup R1b1a2 (R1b-M269) is 78.1% in Cornwall, 62.0% in Leicestershire, and 92.3% in Wales. [19]. In the 1000 genomes GBR cohort, 34 out of 46 male samples belong to haplogroup R1b1a2 making it the most common haplogroup in the UK with 73.9% incidence. Both R1b1a2a1a2c (HI1), and R1b1a2a1a2c1 (HI2) are found once in the GBR of 1000 genomes [20].

The following table lists lists the diagnostic genotype calls for HI1:

SNP/marker	Position	Haplogroup	Ref	Alt	Call
**PF6454,CTS2664**	14416216	R1b1a2	G	A	?
**P257,PF2950,U6**	14432928	G	A	G	-
**PF5896, P244**	14433100	P1	G	A	?
**PF2952,S314,U2**	14577177	G	A	G	-
**PF6541,L52**	14641193	R1b1a2a1a	C	T	?
**F1857,P337,PF5901**	14898094	P1	A	G	+
**L269,PF3135**	14958218	G	C	T	-
**PF2955,L116,S284**	14989721	G	G	C	+
**L402**	15204708	G	G	T	-
**U21**	15204710	G	C	A	-
**L21,M529,S145**	15654428	R1b1a2a1a2c	C	G	+
**S492**	16720013	R1b (investigation)	T	C	-
**S245,Z245**	22200784	R1b (investigation)	C	G	-
**S461,Z290**	28632468	R1b1a2a1a2c	G	C	+

The inference column contains a ``-'' for the ancestral allele, a ``+'' for the derived allele, and a ``?'' for a derived allele which could be due to post-mortem damage.

The calls for HI2 are:

SNP/Marker	Position	Haplogroup	Ref	Alt	Call
**CTS241, DF13,S521**	2836431	R1b1a2a1a2c1,	A	C	+
**S144, L20**	14231292	R1b1a2a1a2b1a1	A	G	-
**PF6454, CTS2664**	14416216	R1b1a2	G	A	?
**U23**	14423856	G	A	G	-
**P257, PF2950, U6**	14432928	G	A	G	-

**P244, PF5896**	14433100	P1	G	A	?
**L382, M3523, PF2951**	14469411	G	A	C	-
**F1794**	14522828	R1b1a2	G	A	?
**S314,PF2952, U2**	14577177	G	A	G	-
**P240, PF5897**	14598808	P1	T	C	?
**U12**	14639427	G	C	A	-
**L52, PF6541**	14641193	R1b1a2a1a	C	T	?
**L32, PF3266,S148,U8**	14692227	G2a2b	C	T	-
**D1857,P337,PF5901**	14898094	P1	A	G	+
**L116, PF2955, S284**	14989721	G	G	C	-
**PF2956, U3**	14993358	G	G	A	-
**P241, M173**	15026424	R1	A	C	+
**PF2957,M201**	15027529	G	T	G	-
**CTS3575**	15037433	R1b1a2	C	G	+
**PF2958**	15086183	G	C	G	-
**L402**	15204708	G	G	T	-
**U21**	15204710	G	C	A	-
**PF3134, U33**	15275200	G	G	C	-
**L21,M529,S145**	15654428	R1b1a2a1a2c	C	G	+
**S492,Z384**	16720013	R1b (investigation)	T	C	-
**Z2542,CTS8221**	17885577	R1b1a2a1a2c1,	C	T	?
**S245,Z245**	22200784	R1b (investigation)	C	G	+
**S461,Z290**	28632468	R1b1a2a1a2c	G	C	+

Contamination Estimates

Contamination estimates using the mitochondrial DNA were done using a comparison against the 1000 genomes database. We identified private or near-private consensus alleles in each individual, requiring the minor allele frequency to be less than 5% in the 1000 genomes cohort of modern DNA. We required the quality score to be at least 50, but did not put a restriction on coverage, since coverage was very high to start with. Furthermore, we excluded the positions where either C or G was the consensus allele, because there is a chance that these are due to post-mortem misincorporations. We did a point estimate of mtDNA contamination following Skoglund, et al. [21]. We assumed independence of the bases, and estimated

$$\hat{c} = \frac{N_{\text{alt}}}{N_{\text{cons}} + N_{\text{alt}}}$$

If no alternative allele was found, the upper confidence limit was calculated as the value of c at P=0.05 in the binomial distribution

$$\binom{N}{k} c^k (1-c)^{N-k}$$

where $k = 0$ and $N = N_{\text{cons}}$. In the cases where no diagnostic sites were found, the contamination could not be estimated. Estimates are listed in Supplementary

Table 3. The comparably high contamination level of HS2 is based one site, 16245, where there are 617 calls supporting T and 79 calls supporting C. HS2* has been calculated by removing this one site. The site 16245 is in the D-loop, or hypervariable region of mitochondrial DNA and it is possible that allele counts on this site are within the natural variation of heteroplasmy. We note that in 1000 genomes cohort there are 10T and 1064C. In addition to the estimates from MT DNA, we used a program called "verifyBamId" [22], which estimates autosomal contamination using the 1000 Genomes reference panel.

Supplementary Note 3 – Rare allele sharing analysis

The main processing for rare allele sharing is described in the Methods section of the paper. Here we provide some additional analysis that we performed to replicate the main results.

Relative allele sharing using Finnish and Spanish outgroups

In addition to the UK10K samples shown in Figure 2 of the main text, we performed a similar analysis using the GBR samples from the 1000 Genomes project. As described in Supplementary Note 4, we identify three subpopulations in the GBR samples, which we can conclusively identify with samples from Cornwall, Kent and the Orkney Islands. In this analysis, we could not use the Dutch and Spanish populations as an outgroup, because the GBR genotypes were called jointly with the Spanish samples from the 1000 Genomes project, while the Dutch samples were called indepently. Therefore, using the Dutch and Spanish populations as outgroups would result in biases towards allele sharing with the Spanish samples. Therefore, we use the Finnish samples from the 1000 Genomes project as outgroup.

Supplementary Figure 5a shows two projections of modern British samples using the Finnish and Spanish populations as outgroup. In the first, we used the same individuals from the UK10K project as used in Figure 2. It shows that the choice of the outgroup (Dutch vs. Finnish) has little influence on our estimate of Anglo-Saxon ancestry in the East of England. In both cases, the samples from the East of England and Kent, respectively, place at about 40% between the Iron Age and the Anglo-Saxon samples. Expectedly, in this projection using the Finnish outgroup, the samples from the Orkney islands share substantially more rare alleles with the Finnish than do the other groups from the GBR samples (Kent and Cornwall) and all three groups from the UK10K project.

Projecting UK10K samples directly onto ancient samples

While the results shown Figure 2 in the main text and in Supplementary Figure 5a above are based on allele sharing with outgroup populations, we also tried a more direct approach of comparing allele sharing with Anglo-Saxon vs. Iron Age samples. Here we took the entire TwinsUK data set from UK10K (with genotype calls provided by UK10K, cite), consisting of 1854 individuals from across the UK, as a reference panel and computed allele sharing of each ancient sample with subpopulations from Wales, East England and Scotland, using all variants up to allele count 37 (1%) in the full data set. In this case, because we had to normalize out coverage differences between the ancient samples, we divided the sharing counts for each ancient sample by the number of shared variants with TwinsUK with allele counts 37 through 370 (1%-10%). We then computed for each TwinsUK sample the mean normalized sharing count with the Iron Age group (H1, H2 and L) and with the Anglo-Saxon era group (HS1, HS2, O1 and O2). We did the same calculation for each ancient individual, by first removing that individual from the two groups above and comparing to the rest of each group. We include samples O3 and O4 for comparison, but they were not used to compute the mean and standard deviation shown in the red Gaussian curve (Supplementary Figure 5b).

We do not try to estimate an Anglo-Saxon component from this analysis because the noise is much stronger than the signal, but we note that the results here are qualitatively consistent with the analyses using outgroups, in particular with the East English samples being somewhat closer to the Anglo-Saxon samples than the groups from Wales and Scotland.

Estimating the Anglo-Saxon component in modern England

Supplementary Table 4 summarizes our estimates of the Anglo-Saxon component in the modern British population, using different outgroup populations (Dutch and Finnish vs. Spanish) and different British populations as test cases. We include both the case with and without HS3 as a member of the Anglo-Saxon group. O3 and O4 are always excluded because they seem admixed or of non-Anglo-Saxon ancestry (see Figure 2 in the main text).

The values and standard deviations in Supplementary Table 4 are the relative sharing fraction of the group indicated in column 2, using the outgroups indicated in column 4. The second-last column gives the estimate of the Anglo-Saxon component in that group using the simple formula

$$\text{Fraction} = \frac{V - I}{S - I},$$

Where V is the value of the modern-British group (e.g. from Kent), I is the value for the Iron Age group, and S is the value for the Anglo-Saxon group. The standard deviation of the fraction is computed using the standard error propagation:

$$\Delta\text{Fraction} = \sqrt{\left(\frac{\Delta V}{S - I}\right)^2 + \Delta I^2 \left(\frac{V - S}{(S - I)^2}\right)^2 + \left(\frac{\Delta S(V - I)}{(S - I)^2}\right)^2}$$

We obtain very consistent results for the South and East of England (highlighted in Supplementary Table 4), using different outgroups and different sample sets. The 1000 Genomes group from Kent and the UK10K samples from the East of England have on average an Anglo-Saxon component of 38% or 37% respectively, with a large spread of up to 21%, which reflects variability among the samples. Samples from Cornwall and Wales have consistent results around 30%, again with a large spread. The Scottish samples from UK10K, in contrast have a similarl Anglo-Saxon component as Wales when using the Dutch outgroup, but a lower component when using the Finnish outgroup. We believe that the result using the Dutch outgroup is appropriate, given that it most strongly separates Anglo-Saxon from Iron Age samples. When excluding sample HS3 from the Anglo-Saxon group, this group gets more defined and further away from the modern and Iron Age samples, resulting in a lower estimate of the Anglo-Saxon component, of around 32-35% in East and Kent samples, depending on the outgroup.

Supplementary Note 4 – Population substructure in the GBR samples from the 1000 Genomes Project

The GBR samples from the 1000 Genomes Project [20] were collected from three sites: Kent, Cornwall and the Orkney Islands. We counted doubleton mutations, i.e. mutations with allele count 2, shared by only two GBR individuals, and generated a count matrix for all pairs of samples (Supplementary Figure 4a). The three subpopulations generate a visible pattern in shared doubletons. The matrix shows that the GBR samples are ordered with respect to sampling location and that they fall into three distinct clusters of 27, 28 and 36 individuals, respectively. In particular the first and third cluster exhibits notable excess allele sharing within the cluster, reflecting relatively strong genetic drift in comparison with the second cluster.

We selected the overlap of SNPs with the Human Origins data set [23,24], and generated a PCA plot of all GBR samples projected onto selected European samples (Supplementary Figure 4b). The PCA shows that the first cluster (GBR1) falls more closely with the Orcadian samples from the Human Origins data set than the other two clusters, so we conclude that the first cluster contains the samples from the Orkney Islands.

From the PCA we cannot infer which of the second and third cluster is sampled from Kent and which from Cornwall. A recent publication on British population structure [25] shows that the population from Cornwall is relatively drifted and internally well defined, which suggests that GBR3 is Cornwall and GBR2 is Kent. Furthermore, as shown in Supplementary Note 5, we used rarecoal to find the best fitting phylogeny of 5 European populations plus each of the three GBR clusters separately, and find that the second cluster forms a clade with the Dutch population, while the third cluster forms an outgroup to the rest of Northern Europe. Given the known Anglo-Saxon influence from the Dutch and German coast into the South East of England, we conclude that the second cluster contains the samples from Kent, and the third cluster contains the samples from Cornwall.

Supplementary Note 5 – Rarecoal Analysis

Rarecoal program

The rarecoal method (Supplementary Note 6) is implemented in a command line tool called "rarecoal", and available on https://github.com/stschiff/rarecoal. This command has several subcommands that are documented in detail on the github-webpage, and of which the following are relevant to this analysis:

- "rarecoal maxl": This command finds the maximum likelihood estimates for all parameters specified in a model. This tool performs a greedy search using the Nelder-Mead-Simplex optimization method and should only be used to get a preliminary estimate of the maximum.
- "rarecoal mcmc": This command performs a Markov-Chain Monte-Carlo simulation on the likelihood function to find the local optimium and get posterior distribution confidence intervals for each parameter. This program will automatically perform a burnin phase which will take as long as needed to find the local maximum, and then perform 1000 MCMC iterations to obtain the confidence intervals
- "rarecoal find": This command takes an additional population or sample and tries every possible place on an existing tree for that additional branch to merge onto the tree. It will output the maximum likelihood branch point.

All outputs of the programs are scaled. To get real times in generations, scaled times need to be multiplied by $2N_0$, and to get real population sizes, scaled population sizes should be multiplied with N_0. In our case, $N_0 = 20{,}000$.

Testing Rarecoal with simulated data

We defined a simple population-tree, as shown in Figure 3b of the paper. We used the SCRM simulator [26] with the following command line to simulate 20 chromosomes of 100Mb:

```
scrm 1000 1 -l 100000 -t 100000 -r 80000 100000000 -I 5
200 200 200 200 200 -ej 0.00125 2 1 -ej 0.0025 4 3 -ej
0.00375 5 3 -ej 0.005 3 1 -en 0.00000001 1 0.1 -en
0.00000002 2 2.0 -en 0.00000003 3 1.0 -en 0.00000004 4
5.0 -en 0.00000005 5 10.0 -en 0.00125001 1 1.0 -en
0.0025001 3 0.5 -en 0.00375001 3 0.8 -en 0.005001 1 1.0
```

The tree topology of this tree is (((0, 1), ((2, 3)), 4)), with branches ordered left to right as in Figure 3b in the main text. We first obtained maximum likelihood estimates of only the split times, and a globally fixed population size. Note: all times are scaled with $2N_0$ (not $4N_0$ as in the command line above), and all population sizes are scaled by N_0.
This first round of maximization using "rarecoal maxl" is summarized in the following table:

Parameter	True value	Initial value	Estimate
$t_{(0,1)}$	0.0025	0.001	0.00271
$t_{(2,3)}$	0.005	0.002	0.00242

$t_{((2,3),4)}$	0.0075	0.003	0.00452
$t_{(((0,1),(2,3),4)}$	0.01	0.004	0.00592
N_{global}	1	1	0.859

We then used these estimates as starting point for the full model optimization, with separate population size estimates in each internal and leaf-branch of the tree. We denote the population size parameters with N, using as subscript the subtree of the node below that branch. The results are summarized in the following table, including confidence intervals for each parameter as obtained by "rarecoal mcmc":

Parameter	True Value	Median Estimate	95% CI
$t_{(0,1)}$	0.0025	0.002790	(0.002773, 0.00284)
$t_{(2,3)}$	0.005	0.005078	(0.00506, 0.00510)
$t_{((2,3),4)}$	0.0075	0.00779	(0.00776, 0.0078)
$t_{(((0,1),(2,3),4)}$	0.01	0.00979	(0.00973, 0.00982)
N_0	0.1	0.1055	(0.1052, 0.1057)
N_1	2	2.38	(2.35, 2.42)
N_2	1	1.006	(1.003, 1.01)
N_3	5	5.08	(5.03, 5.14)
N_4	10	10.60	(10.48, 10.73)
$N_{(0,1)}$	1	0.90	(0.89, 0.91)
$N_{(2,3)}$	0.5	0.52	(0.51,0.53)
$N_{((2,3),4)}$	0.8	0.64	(0.63, 0.65)
$N_{(((0,1),(2,3),4)}$	1	0.98	(0.97, 0.99)

Simulating a lower sample size

In the real data, we have diploid sample sizes of about 100 for the Finnish, British, Spanish, Italian and Dutch samples, and only 20 for the Danish population. To see whether the lower sample size in the Danish population creates a bias on the estimates, we generated a simulation similar to the one above, but with only 20 samples for the last population. The command line was

```
scrm 940 1 -l 100000 -t 100000 -r 80000 100000000 -I 5
200 200 200 200 40 -ej 0.00125 2 1 -ej 0.0025 4 3 -ej
0.00375 5 3 -ej 0.005 3 1 -en 0.00000001 1 0.1 -en
0.00000002 2 2.0 -en 0.00000003 3 1.0 -en 0.00000004 4
5.0 -en 0.00000005 5 10.0 -en 0.00125001 1 1.0 -en
0.0025001 3 0.5 -en 0.00375001 3 0.8 -en 0.005001 1 1.0
```

The MCMC analysis on this dataset was started from the same values as in the analysis of the full simulation, and yielded the following results:

Parameter	True Value	Median Estimate	95% CI
$t_{(0,1)}$	0.0025	0.00279	(0.00277, 0.00280)
$t_{(2,3)}$	0.005	0.005	(0.00598, 0.00503)
$t_{((2,3),4)}$	0.0075	0.00814	(0.00809, 0.00816)
$t_{(((0,1),(2,3),4)}$	0.01	0.00961	(0.00958, 0.00966)
N_0	0.1	0.106	(0.105, 0.106)
N_1	2	2.42	(2.38, 2.45)
N_2	1	0.994	(0.98, 1.00)
N_3	5	4.89	(4.84, 4.94)
N_4	10	11.7	(11.0, 12.3)
$N_{(0,1)}$	1	0.87	(0.86, 0.88)
$N_{(2,3)}$	0.5	0.60	(0.59,0.61)
$N_{((2,3),4)}$	0.8	0.445	(0.44, 0.47)
$N_{(((0,1),(2,3),4)}$	1	1.00	(0.99, 1.01)

Again, the estimates are close to the truth, with the exception of $N_{((2,3),4)}$, so the ancestral population size involving the population with the lower sample size. We conclude that the overall tree is not affected from including a population with a much lower sample size, but that population size estimates in internal branches of the tree can be affected by lower sample sizes.

Testing robustness under admixture

We also tested how admixture affected parameter estimates. We simulated three populations under a model shown in Supplementary Figure 7a. We simulated 20 chromosomes of this model under a variety of admixture rates m, using the command line:

```
scrm 600 1 -p 12 -t 100000 -r 80000 100000000 -I 3 200
200 200 -eps 0.00125 2 3 (1-<m>) -ej 0.001875 2 1 -ej
0.00375 3 1 -seed 1
```

We then used "rarecoal mcmc", starting with the true split times and population sizes parameters of the model to estimate parameters for each simulated data set. The results are shown in Supplementary Figure 7b. Under zero admixture, the estimated parameters are very close to the true parameters, but with increasing rates of admixture, some estimates get worse, as expected, since rarecoal does not currently implement admixture. In particular, the population size of the recipient population of the admixture event (P1) is overestimated, and the older split time (t02) is underestimated. The former effect could be causing the high ancestral population size of the ancestral Spanish/Italian population (see below).

Learning the European population tree

In the following, we use three letter abbreviations for the populations studied here, which are

- FIN: Finnish from 1000 Genomes [20]

- GBR: British from 1000 Genomes
- IBS: Spanish from 1000 Genomes
- TSI: Italian from 1000 Genomes
- NED: Dutch from the GoNL data set [27]
- DMK: Danish from the GenomeDK project [28]

We started with three populations (FIN, IBS, NED) and tested all three possible tree topologies for these populations, with one global population size. The best tree, obtained via "rarecoal maxl" is ((FIN, NED), IBS) with scaled split times 0.0039 and 0.006, and a global population size of 2.3.

We then added the Danish branch and tested every possible point in the tree to join. The maximum likelihood point to join, obtained via "rarecoal find" was the Dutch branch at time 0.0028, resulting in the topology ((FIN, (NED, DMK)), IBS). We then maximized split times and a global population size on that tree using "rarecoal maxl" and found split times 0.003, 0.0038 and 0.006 with a global population size of 2.34.

Next, we added the TSI as additional population to the tree and first again checked every possible point in the tree to merge. We found that the maximum likelihood point in the tree was - surprisingly - on the Danish branch at an extremely recent time 0.0001. The second highest hit was a merge onto the Spanish branch at time 0.0023. We note that the TSI/DMK branch point may not reflect the optimal tree topology, because the branch-point search is not searching through the full space of models including individual population sizes in each branch, as is MCMC. Instead of performing MCMC on this candidate topology (TSI branching onto the DMK branch), we immediately tried the second highest merge-point with the TSI/IBS merge-point, resulting a topology ((FIN, (NED, DMK)), (IBS, TSI)). Using this candidate topology and the previous parameters as initial parameters, we then again estimated maximum likelihood parameters for this five-population tree and found parameters summarized in the following table:

Parameter	Estimate
$t_{(\mathrm{NED,\ DMK})}$	0.0024
$t_{((\mathrm{FIN,\ (NED,\ DMK)})}$	0.0032
$t_{(\mathrm{IBS,\ TSI})}$	0.0049
$t_{((\mathrm{FIN,\ (NED,\ DMK)),\ (IBS,\ TSI)})}$	0.0062
N_{global}	3.15

We then allowed for separate population sizes within each branch of the tree and inferred parameters using maximization and subsequent MCMC. The results for the median estimates after MCMC are:

Parameter	Estimate
$t_{(\mathrm{NED,\ DMK})}$	0.0039
$t_{((\mathrm{FIN,\ (NED,\ DMK)})}$	0.004

$t_{(IBS, TSI)}$	0.0054
$t_{((FIN, (NED, DMK)), (IBS, TSI))}$	0.0064
N_{FIN}	0.53
N_{IBS}	8.23
N_{TSI}	6.89
N_{NED}	8.37
N_{DMK}	1.87
$N_{(NED, DMK)}$	1.05
$N_{((FIN, (NED, DMK))}$	0.94
$N_{(IBS, TSI)}$	983.25
$N_{((FIN, (NED, DMK)), (IBS, TSI))}$	2.00

Finally, we added the British population branch, by first again trying every possible point for it to merge into the tree. We found that the most likely point to merge was on the Netherland branch at time 0.0007. We used this as a starting point for another round of parameter estimation, and found that the resulting tree had two suspiciously close population splits, with a star-like split of GBR, NED and FIN. We therefore changed the topology and tried whether merging the GBR population into the ancestral (FIN, (NED, DMK))-branch would give a higher likelihood. Indeed this was the case, so the best fitting tree topology is (((FIN, (NED, DMK)),GBR),(TSI, IBS)). The final parameter estimates are:

Parameter	Estimate	95% CI
$t_{(NED, DMK)}$	0.00413	(0.00412, 0.00415)
$t_{((FIN, (NED, DMK))}$	0.00438	(0.00436, 0.00440)
$t_{((FIN, (NED, DMK)), GBR)}$	0.00449	(0.00447, 0.00451)
$t_{(IBS, TSI)}$	0.00174	(0.00168, 0.00184)
$t_{(((FIN, (NED, DMK)), GBR), (IBS, TSI))}$	0.00601	(0.00599, 0.00603)
N_{FIN}	0.60	(0.6, 0.6)
N_{GBR}	4.87	(4.82, 4.94)
N_{IBS}	3.93	(3.8, 4.12)
N_{TSI}	3.26	(3.16, 3.42)
N_{NED}	9.96	(9.7, 10.2)
N_{DMK}	1.95	(1.91, 1.99)
$N_{(NED, DMK)}$	0.57	(0.55, 0.60)
$N_{((FIN, (NED, DMK))}$	0.71	(0.67, 0.76)
$N_{((FIN, (NED, DMK)), GBR)}$	0.64	(0.64, 0.64)
$N_{(IBS, TSI)}$	997	(990, 1000)
$N_{(((FIN, (NED, DMK)), GBR), (IBS, TSI))}$	1.02	(1.02, 1.02)

Since all split times are well separated considering their confidence interval, we conclude that this model represents the maximum likelihood model. If the topology was suboptimal, then the maximum likelihood result would involve star-like branch-points, with split times falling within each others confidence intervals. We also tried whether the high ancestral population size of the IBS/TSI

branch was a sub-optimal local maximum, by restarting the MCMC from a lower population size and an earlier IBS/TSI split time. This resulted in similar estimates as the ones presented above, so we conclude that this tree is the maximum likelihood tree, which is shown in Supplementary Figure 8a. The extremely high Spanish/Italian ancestral population size could an artifact of population admixture, as shown in the previous section.

Substructure in the GBR samples

As we have described in Supplementary Note 4, there is a clear substructure within the GBR samples, and so we tested each population separately with the other 5 populations. The results are shown in Supplementary Figure 8b. We first used the same tree topology as inferred for the complete GBR set above and found that it fitted well for the Orkney and Cornwall clusters, but not for the Kent cluster. We then changed the tree topology such that the Kent population was allowed to merge into the Dutch branch before other splits and obtained a significantly better fit. This suggests that the Kent population in the South of England is significantly closer to the Dutch population than both the Cornwall and Orkney group, consistent with Anglo-Saxon immigrations. This result also confirms that the second cluster in the GBR are the Kent samples, and the third cluster are the Cornish samples (see Supplementary Note 4).

Mapping individuals onto the tree

For mapping the ancient individuals onto the tree, we first generate data sets consisting of all the European individuals that went into learning the European tree, plus one additional individual. We then use the program "rarecoal find" to compute the likelihood for all branch points of the additional branch onto the tree. We vary the merge point of that additional population, over all leaf- and internal branches of the European tree, with a discretized time interval of scaled time 0.0001. In "rarecoal find", we set the options "--conditionOn" and "—minAf" to restrict the likelihood computation on sites at which the additional sample has a derived allele, and in which at least one other individual in the Reference data set has the derived allele.

We tested this approach with individuals from the 1000 Genomes project [20], which for this analysis were taken out of the reference set of FIN, GBR, IBS and TSI samples. As seen in Supplementary Figure 11, all the FIN, IBS and TSI samples fall expectedly onto the tip of their respective population branch. For the GBR individuals from Cornwall, we find that they map onto the branch of the Cornish population, as expected. When mapping individuals from Kent and Orkney, we find that they fall onto the common ancestor of all Northern European populations, similarly as the Iron Age samples.

When we use the European tree with the Kent population as British population branch, the mapping of modern samples looked different (Supplementary Figure 12). While for the FIN, IBS and TSI samples, mapping still works as expected, GBR samples from Kent do not fall onto the Kent branch. Also, one sample from Cornwall maps to the Spanish branch. The most likely explanation is that the Kent population is an admixed population and hence poorly modeled by a tree without gene flow or admixture. While the maximum-likelihood tree still places

the Kent branch closest to the Dutch population, individuals from Kent are of admixed European ancestry and hence map most likely into the ancestral branch of Northern European populations. The fact that one of the two Cornish samples maps onto the Spanish branch suggests that some Cornwall samples are genetically closer to Southern Europe than to Kent, again reflecting a more complex European history than can be modeled using simple trees.

In conclusion, we find that our approach of mapping individuals into the European tree works well for a tree with the Cornish population as British population branch, which are a relatively defined group in contrast to the samples from Kent, which have little private allele sharing (see Supplementary Figure 4a) and a large population size. In addition, it may be too admixed to be put into a simple tree phylogeny.

Supplementary Note 6 - Rarecoal Theory

The rarecoal coalescent framework

Rarecoal is a coalescent framework for rare alleles. We define rare alleles roughly by requiring i) the allele count of the derived mutation to be small, typically not larger than 10, and ii) the total number of samples to be much larger, say 100 or more. The idea is to provide a general approach of computing the joint allele frequency spectrum for rare alleles under an arbitrary demographic model under population splits and population size changes. Migration and admixture will be incorporated in the future.

Definitions

In the following, we compute the probability to observe a pattern of rare alleles seen across multiple populations, given a demographic model. In the simplest case, a demographic model is tree-like and consists of population split times and constant population sizes in each branch of the tree. Time is counted backwards in time, with $t = 0$ denoting the present and $t > 0$ denoting scaled time in the past. We denote the scaled coalescence rate (scaled inverse population size) in population k at time t by $\lambda_k(t) = N_0/N_k(t)$, where $N_k(t)$ is the population size in population k at time t, and N_0 is a scaling constant which we set to $N_0 = 20000$ for modeling human evolution.

We consider a number of P subpopulations. We define a vector $\mathbf{n} = \{n_k\}$ for $k = 1 \dots P$ summarizing the number of sampled haplotypes in each population. We also define vector $\mathbf{m} = \{m_k\}$ as the set of derived allele counts at a single site in each population. As an example, consider 5 populations with 200 haplotypes sampled in each population, and a rare allele with total allele count 3, with one derived allele seen in population 2 and 2 derived alleles seen in population 3. Then we have $\mathbf{n} = \{200, 200, 200, 200, 200\}$ and $\mathbf{m} = \{0, 1, 2, 0, 0\}$.

Looking back in time, lineages coalesce and migrate, so the numbers of ancestral and derived alleles in the past decrease over time. In theory one needs to consider a very large state space of configurations for this process, with one state for each possible number of ancestral and derived lineages in each population. Here we make a major simplification: While we will consider the full probability distribution over the derived lineages, we will consider only the expected number of ancestral alleles over time. Specifically, we define the expected number of ancestral alleles in population k at time t as $\mathbf{a}(t) = \{a_k(t)\}$. For the derived alleles, we define a state $\mathbf{x} = \{x_k\}$ as a configuration of derived lineages in each population. The probability for state $\mathbf{x}$ at time t is defined by $b(\mathbf{x}, t)$.

Coalescence

We now consider the evolution of the two variables $a(t)$ and $b(\mathbf{x}, t)$ through time under the standard coalescent. We first introduce a time discretization. We define time points $t_0 = 0, \dots t_T$. Here, $t_T = t_{\max}$ should be far enough in the past to make sure that most lineages have coalesced by then with a high probability. We choose a time patterning that is linear in the beginning and crosses over to an exponentially increasing interval width. Specifically, the patterning follows this equation, inspired by the time discretization in (Li and Durbin, 2011):

$$t_i = \alpha \exp\left(\frac{i}{T} \log\left(1 + \frac{t_{\max}}{\alpha})\right)\right) - \alpha. \tag{1}$$

Here, T is the number of time intervals, and α is a parameter that controls the crossover from linear to exponential scale. In practice, we use $\alpha = 0.01$, $t_{\max} = 20$ and $T = 3044$, which are chosen such that

the initial step width equals one generation (in scaled units with $N_0 = 20000$), and the crossover scale is 400 generations.

Given the number of sampled haplotypes in each population n_k, and the observed number of derived alleles m_k in each population, we initialize our variables as follows:

$$a_k(t=0) = n_k - m_k. \tag{2}$$

for each population k, and

$$b(\mathbf{x}, t=0) = 1 \text{ if } x_k = m_k \text{ for all } k = 1 \dots P \tag{3}$$
$$b(\mathbf{x}, t=0) = 0 \text{ otherwise} \tag{4}$$

Under a linear approximation, we can compute the value of $\mathbf{a}$ at a time point $t + \Delta t$, given the value at time t:

$$a_k(t+\Delta t) = a_k(t)\left(1 - \frac{1}{2}(a_k(t) - 1)\lambda_k(t)\Delta t\right). \tag{5}$$

The factor 1/2 corrects overcounting: any one coalescence takes one of two lineages out, so it should be counted half per participating lineage. We can improve this update equation slightly beyond the linear approximation: In the limit of $\Delta t \to 0$, equation 5 forms a differential equation which can be solved for finite intervals Δt:

$$a_k(t+\Delta t) = \frac{1}{1 + \left(\frac{1}{a_k(t)} - 1\right)\exp\left(-\frac{1}{2}\lambda_k(t)\Delta t\right)}. \tag{6}$$

For the derived alleles, we need to update the full probability distribution $b(\mathbf{x}, t)$:

$$\begin{aligned} b(\mathbf{x}, t+\Delta t) =& b(\mathbf{x}, t)\exp\left(-\sum_k \left(\binom{x_k}{2}\lambda_k(t) + x_k a_k(t)\lambda_k(t)\right)\Delta t\right) \\ &+ \sum_l b(x_1 \dots (x_l+1) \dots x_P, t)\left(1 - \exp\left(\binom{x_l+1}{2}\lambda_l(t)\Delta t\right)\right) \end{aligned} \tag{7}$$

where the first term accounts for the reduction of the probability over time due to derived lineages coalescing among themselves or coalescing with an ancestral lineage, and the second term accounts for the increase from those two processes occurring in states with a higher number of derived lineages. In contrast to the equation for $a(t)$, we cannot solve this as a differential equation and will only use this linear approximation in Δt.

Population Splits

We now consider the case where a single ancestral population splits into two separate groups at some point in time. When modelling this in a coalescent framework, we have to look at this backward in time, and thus a population split is viewed as two separate populations that join into one ancestral population at some point in time. We consider a population join backward in time from population l into population k. For the non-derived lineages, this means that after the join, population k contains the sum of lineages from population k and l:

$$a'_k(t) = a_k(t) + a_l(t) \tag{8}$$
$$a'_l(t) = 0 \tag{9}$$

where the primed variable marks the variable after the event, which will then be used as the basis for the next coalescence update.

For the derived lineages, we need to sum probabilities in the correct way. We first define a transition function that changes a state before the join to new states after the join:

$$\mathbf{x}' = J(\mathbf{x}), \tag{10}$$

where

$$J((\dots x_k \dots x_l \dots)) = (\dots (x_k + x_l) \dots 0 \dots) \tag{11}$$

We can then define the join itself as a sum over all states before the join that give rise to the same state after the join:

$$b'(\mathbf{x}', t) = \sum_{\mathbf{x}, J(\mathbf{x}) = \mathbf{x}'} b(\mathbf{x}, t) \tag{12}$$

The likelihood of a configuration of rare alleles

Eventually we want to compute the probability for a given configuration ($\mathbf{n}$, $\mathbf{m}$) observed in the present. This probability is equal to the probability that a) all derived lineages coalesce before any of them coalesces to any ancestral-allele lineage, and b) that a mutation occurred on the single lineage ancestral to all derived lineages.

We define a singleton state $\mathbf{s}^k$ to be the state in which only $x_k = 1$ and $x_l = 0$ for $l \neq k$. We accumulate the total probability for a single derived lineage:

$$d(t + \Delta t) = d(t) + \sum_k b(\mathbf{s}^k)\Delta t. \tag{13}$$

Then the likelihood of the configuration under the model is

$$L(\mathbf{n}, \mathbf{m}) = \mu d(t_{\max}) \prod_{k=1}^{P} \binom{n_k}{m_k}, \tag{14}$$

which is the total probability of a mutation occurring on a single derived lineage, times the number of ways that $\mathbf{m}$ derived alleles can be drawn from a pool of $\mathbf{n}$ samples. Note that $d(t_{\max})$ depends on $\mathbf{n}$, $\mathbf{m}$ and the demographic parameters, which we have omitted for brevity so far.

Parameter estimation

The above framework presents a way to efficiently compute the probability of observing a distribution of rare alleles, $\mathbf{m}$ for a large number of samples $\mathbf{n}$ in multiple subpopulations, given a demographic model. We can summarize the full data as a histogram of rare allele configurations. We denote the ith allele configuration by $\mathbf{m}_i$ and the number of times that this configuration is seen in the data by $N(\mathbf{m}_i)$. We then write

$$\mathcal{L}(\{N(\mathbf{m}_i)\}|\Theta) = \prod_i L(\mathbf{m}_i|\Theta)^{N(\mathbf{m}_i)}, \tag{15}$$

where we have introduced a meta-parameter Θ that summarizes the entire model specification (population split times and branch population sizes), and we have made the dependency of L (eq. 14) on Θ explicit. For brevity we have omitted the sample sizes $\mathbf{n}$. For numerical purpose, we always consider the logarithm of this:

$$\log \mathcal{L}(\{N(\mathbf{m}_i)\}|\Theta) = \sum_i N(\mathbf{m}_i) \log L(\mathbf{m}_i|\Theta). \tag{16}$$

The sum in equation 16 comprises all possible configurations in the genome, in principle. In practice, we only explicitly compute it for configurations between allele count 1 and 4, and replace the rest of the counts with a bulk probability:

$$\log \mathcal{L}(\{N(\mathbf{m}_i)\}|\Theta) = \sum_i I(\mathrm{AC}(i)) N(\mathbf{m}_i) \log L(\mathbf{m}_i|\Theta) + N_{\mathrm{other}} \log L_{\mathrm{other}}(\Theta), \tag{17}$$

where the indicator function $I(\mathrm{AC}(i))$ gives 0 if the allele count is between 1 and 4, and 0 otherwise. The bulk count N_{other} simply counts up sites with either no variant or variants with allele count larger than 4. The bulk probability is simply:

$$L_{\mathrm{other}}(\Theta) = 1 - \sum_i (1 - I(\mathrm{AC}(i)) L(\mathbf{m}_i|\Theta), \tag{18}$$

With a given population tree and a given histogram of allele configuration counts $N(\mathbf{m}_i)$, we implemented numerical optimizations over the parameters Θ to find the maximum likelihood parameters, and MCMC to estimate the posterior distributions for all parameters given the data. We usually first search for the maximum with the optimization method, which is much faster than MCMC, and then use MCMC to explore the distribution around that maximum.

Implementation

We implemented this method in the Haskell programming language as a program called “rarecoal”, available from github at `https://github.com/stschiff/rarecoal`.

Supplementary References

1 Clarke, A. C., Spoerry, P. & Leith, S. Cambridgeshire: Part II Excavations at Hinxton Hall and the Genome Campus, 1993-2011: Anglo-Saxon to Medieval Settlement. *East Anglian Archaeology Monograph* (Forthcoming).

2 Lyons, A. Cambridgeshire Part I: Excavations at the Genome Campus 1993-2014: Ritual and Farming in the Cam Valley. *East Anglian Archaeology Monograph* (Forthcoming).

3 Bayliss, A., Hines, J., Hoilund-Nielsen, K., McCormac, G. & Scull, C. *Anglo-Saxon Graves and Grave Goods of the 6th and 7th Centuries AD (Society for Medieval Archaeology: Monograph)*. (Maney Publishing, 2013).

4 Sayer, D. & Dickinson, S. D. Reconsidering obstetric death and female fertility in Anglo-Saxon England. *World Archaeology* **45**, 285-297 (2013).

5 Lucy, S. *The Anglo-Saxon way of death: burial rites in early England*. (Sutton Pub Limited, 2000).

6 Stoodley, N. *The spindle and the spear: a critical enquiry into the construction and meaning of gender in the early Anglo-Saxon burial rite*. Vol. 208 (British Archaeological Reports, 1999).

7 Sayer, D. Death and the family Developing generational chronologies. *Journal of Social Archaeology* **10**, 59-91 (2010).

8 Williams, H. M. & Sayer, D. Halls of mirrors: death & identity in medieval archaeology. (2009).

9 Härke, H. Anglo-Saxon immigration and ethnogenesis. *Medieval Archaeology* **55**, 1-28 (2011).

10 Meaney, A. L. *A gazetteer of early Anglo-Saxon burial sites*. (London: Allen & Unwin, 1964).

11 Taylor, A., Duhig, C. & Hines, J. in *Proc Cambridge Antiq Soc.* 57-90.

12 Mortimer, R., Sayer, D. & Wiseman, R. in *Life on the edge: Social, Political and Religious Frontiers in Early Medieval Europe.* (ed S. Semple) (In Press).

13 Sayer, D. 'Sons of athelings given to the earth': 1 Infant Mortality within Anglo-Saxon Mortuary Geography. *Medieval Archaeology* **58**, 78-103 (2014).

14 van Oven, M. & Kayser, M. Updated comprehensive phylogenetic tree of global human mitochondrial DNA variation. *Hum Mutat* **30**, E386-394, doi:10.1002/humu.20921 (2009).

15 Zheng, H. X., Yan, S., Qin, Z. D. & Jin, L. MtDNA analysis of global populations support that major population expansions began before Neolithic Time. *Sci Rep* **2**, 745, doi:10.1038/srep00745 (2012).

16 Behar, D. M. *et al.* A "Copernican" reassessment of the human mitochondrial DNA tree from its root. *Am J Hum Genet* **90**, 675-684, doi:10.1016/j.ajhg.2012.03.002 (2012).

17 Brandt, G. *et al.* Ancient DNA reveals key stages in the formation of central European mitochondrial genetic diversity. *Science* **342**, 257-261, doi:10.1126/science.1241844 (2013).

18 Wei, W. *et al.* A calibrated human Y-chromosomal phylogeny based on resequencing. *Genome Res* **23**, 388-395, doi:10.1101/gr.143198.112 (2013).

19 Balaresque, P. *et al.* A predominantly neolithic origin for European paternal lineages. *PLoS Biol* **8**, e1000285, doi:10.1371/journal.pbio.1000285 (2010).
20 1000 Genomes Project Consortium *et al.* A global reference for human genetic variation. *Nature* **526**, 68-74, doi:10.1038/nature15393 (2015).
21 Skoglund, P. *et al.* Genomic diversity and admixture differs for Stone-Age Scandinavian foragers and farmers. *Science* **344**, 747-750, doi:10.1126/science.1253448 (2014).
22 Jun, G. *et al.* Detecting and estimating contamination of human DNA samples in sequencing and array-based genotype data. *Am J Hum Genet* **91**, 839-848, doi:10.1016/j.ajhg.2012.09.004 (2012).
23 Haak, W. *et al.* Massive migration from the steppe was a source for Indo-European languages in Europe. *Nature* **522**, 207-211, doi:10.1038/nature14317 (2015).
24 Lazaridis, I. *et al.* Ancient human genomes suggest three ancestral populations for present-day Europeans. *Nature* **513**, 409-413, doi:10.1038/nature13673 (2014).
25 Leslie, S. *et al.* The fine-scale genetic structure of the British population. *Nature* **519**, 309-314, doi:10.1038/nature14230 (2015).
26 Staab, P. R., Zhu, S., Metzler, D. & Lunter, G. scrm: efficiently simulating long sequences using the approximated coalescent with recombination. *Bioinformatics* **31**, 1680-1682, doi:10.1093/bioinformatics/btu861 (2015).
27 Genome of the Netherlands Consortium. Whole-genome sequence variation, population structure and demographic history of the Dutch population. *Nat Genet* **46**, 818-825, doi:10.1038/ng.3021 (2014).
28 Besenbacher, S. *et al.* Novel variation and de novo mutation rates in population-wide de novo assembled Danish trios. *Nat Commun* **6**, 5969, doi:10.1038/ncomms6969 (2015).
29 Li, H. & Durbin, R. Inference of human population history from individual whole-genome sequences. *Nature* **475**, 493-496, doi:10.1038/nature10231 (2011).
